# Multimodal parameters for the diagnosis of putative primaries of cancer of unknown primary

**DOI:** 10.1016/j.isci.2026.115558

**Published:** 2026-04-01

**Authors:** Shuangyue Pan, Jinyan Wang, Jing Liu, Simin He, Xin Liu, Xiaowei Zhang, Shiyu Jiang, Yanli Wang, Xiaoyan Zhou, Shaoli Song, Liangping Zhou, Haiming Li, Jianfeng Luo, Zhiguo Luo, Silong Hu, Hongxia Wang, Qifeng Wang, Xichun Hu

**Affiliations:** 1 Department of Medical Oncology, Fudan University Shanghai Cancer Center, No. 270, Dong An Road, Shanghai 200032, China; 2 Department of Oncology, Shanghai Medical College of Fudan University, No. 270, Dong An Road, Shanghai 200032, China; 3 Department of Pathology, Fudan University Shanghai Cancer Center, No. 270, Dong An Road, Shanghai 200032, China; 4 Department of Radiology, Fudan University Shanghai Cancer Center, No. 270, Dong An Road, Shanghai 200032, China; 5 Department of Nuclear Medicine, Fudan University Shanghai Cancer Center, No. 270, Dong An Road, Shanghai 200032, China; 6 Department of Biostatistics, School of Public Health, Fudan University, No. 138, Medical College Road, Shanghai 200032, China

**Keywords:** oncology, diagnostics

## Abstract

The Fudan cancer of unknown primary (CUP)-001 trial showed that the 90-gene assay-guided site-specific therapy (SST) can improve the survival of patients with CUP. A multidisciplinary team (MDT) for CUP explored whether multimodal parameters could improve the diagnosis of the putative primary site (PPS). The MDT adjudicated that 64 (49.2%), 22 (16.9%), 8 (6.2%), 14 (10.8%), and 4 (3.1%) patients had clues in pathology/immunohistochemistry (IHC), sentinel lymph nodes, Batson plexus, serum tumor marker patterns, and next-generation sequencing (NGS), respectively. The tumor marker was discarded because the PPS suggested by it had a less than 50% concordance rate with the PPS by the 90-gene assay or pathology/IHC. Only five patients (3.8%) had PPS diagnoses revised by the MDT. 113 (86.9%) patients were stageable as per the MDT, and the M1 stage was an independent prognostic factor for survival. In conclusion, the integration of the 90-gene assay with other multimodal parameters helps to establish a more reasonable, prognosis-related PPS diagnosis.

## Highlights

• Diagnosis of cancer of unknown primary depends on multimodal parameters
• Multidisciplinary team finalizes the diagnosis of putative primary of cancer of unknown primary
• TNM staging shows prognostic value in cancer of unknown primary
• Patients with M0 diseases may have the chance of being amenable to radical local treatment

## Graphical abstract

**Figure:**
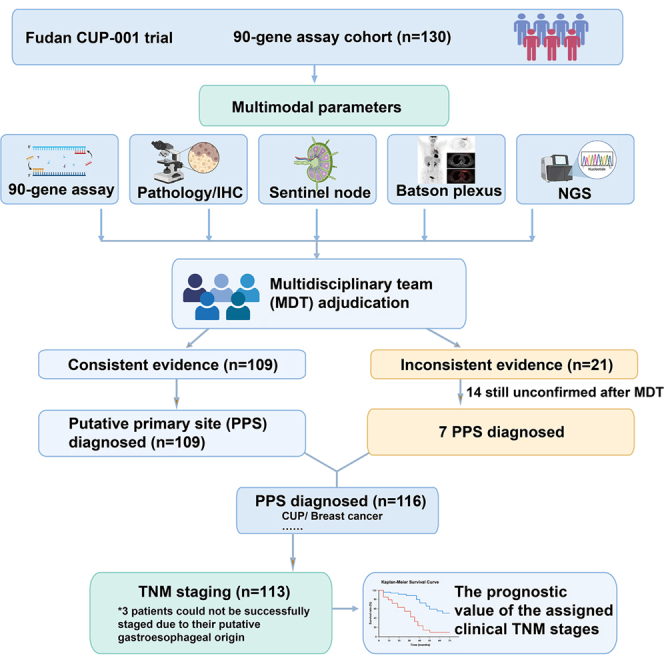

## Introduction

Cancer of unknown primary (CUP) represents a heterogeneous group of cancers where the anatomical site of origin remains unidentified despite comprehensive diagnostic evaluation.^1^^,^^2^ CUP poses significant diagnostic and therapeutic challenges due to its biological heterogeneity and poor response to standard chemotherapy, and about 80% of them have a high mortality rate.^3^^,^^4^ In the Fudan CUP-001 randomized trial (NCT03278600),^5^ the site-specific therapy (SST) guided by the 90-gene tissue of origin test, one gene expression profiling (GEP) assay, significantly improved progression-free survival (PFS). The median PFS for the SST group was 9.6 months, compared to 6.6 months for the empirical chemotherapy (EC) group, demonstrating both statistical and clinical significance. Moreover, the median overall survival (OS) of the SST group was 28.2 months, while the EC group was 19 months, indicating a clinically meaningful extension of survival. This randomized trial has been considered to be practice-changing for patients with CUP.^6^

The 90-gene assay (Canhelp-Origin test) evaluates the similarity of tissue of origin relevant gene-expression patterns between each specimen and 21 types of known primary solid tumors.^7^^,^^8^ The tumor type with the highest similarity score is judged as the putative primary site (PPS).^7^^,^^8^ Results with a similarity score cutoff of more than 45% (the 15^th^ percentile of scores in validation data) were established in the primary known cancers, as predictions above this threshold were considered to have a stronger association with the tissue of origin.^8^ Based on these results, it was approved by the National Medical Products Administration (NMPA) to determine the tissue of origin of cancerous lesions in July, 2022, which provides an objective tool for the diagnosis of CUP. Since CUP may retain the molecular signature of their predicted primary origin,^9^^,^^10^ extending the management approaches used for the primary known cancers to patients with CUP can contribute to the accessibility of therapies for this disease, and possibly similar survival.^1^^,^^11^^,^^12^

CUP is a diagnosis of exclusion, requiring the elimination of any clinically suspected lesions as the primary site.^13^ Immunohistochemistry (IHC) plays a central role in identifying the primary site by assessing the expression patterns of tumor lineage markers and cell-type specific markers.^14^^,^^15^^,^^16^ However, only about 30% of patients with CUP can be assigned the PPSs based on conventional pathology and IHC.^3^^,^^17^ GEP has an accuracy of 80%–90%, while 10%–20% of predicted PPSs were inaccurate, mostly because of rare cancers and gene expression overlaps.^18^ Clinically, some characteristic symptoms, tumor marker patterns, sites, distribution, and metabolism of positive lymph nodes, and the solitary or limited bone metastasis, all have been shown to suggest the tissue of origin of CUP.^19^^,^^20^^,^^21^^,^^22^ Moreover, fluorodeoxyglucose (FDG) positron emission tomography-computed tomography (PET-CT) has a role in tracking primary sites of CUP, but it is not recommended in routine practice.^23^ However, through thorough consults between members of the multidisciplinary team (MDT), a whole body FDG PET-CT can provide not only the anatomic information of suspected lesions, but also metabolic information, as well as the presence or absence of clues in sentinel lymph nodes and bone metastasis mediated by the Batson plexus (a valveless venous network that enables regional tumor spread from abdominal, thoracic, or pelvic organs to adjacent axial bones without systemic spreading).^19^^,^^20^

In this study, in the 130 patients from the Fudan CUP-001 trial who had had interpretable 90-gene assay and FDG PET-CT results, a specialized MDT in our center prospectively assessed all possible clinicopathologic parameters as clues suggestive of PPS and compared them with the 90-gene assay and pathology/IHC; tried to clinically stage all patients with CUP based on PPS predicted by the 90-gene assay results as well as adjudicated by the MDT; and explored the prognostic value of the designated clinical TNM stages.

## Results

### Baseline characteristics

**Figure 1:**
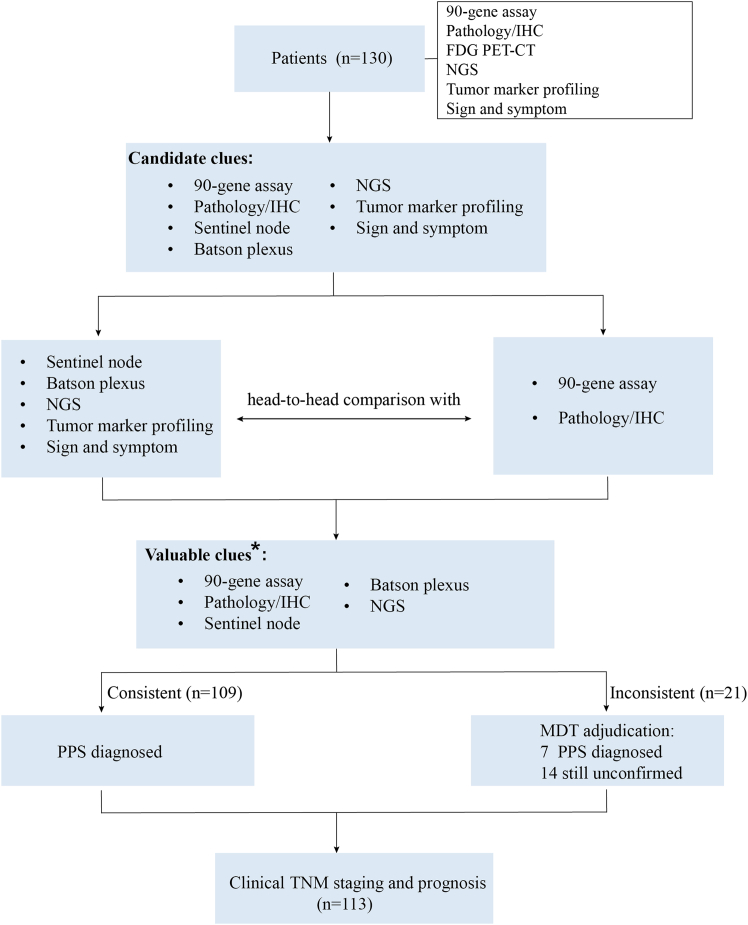
Flowchart for MDT assessment of multimodal parameters A specialized multidisciplinary CUP team prospectively evaluated multimodal parameters in the 130 patients from the Fudan CUP-001 trial and found that pathology/IHC, sentinel node, Batson plexus, and NGS could provide supportive evidence to the 90-gene assay for deducing PPS. The MDT integrated these valuable clues to diagnose PPS of patients with CUP, clinically staged patients with CUP, and explored the prognostic value of the assigned clinical TNM stages. PPS, putative primary site. MDT, multidisciplinary team; CUP, cancer of unknown primary; IHC, immunohistochemistry; FDG PET-CT, fluorodeoxyglucose positron emission tomography-computed tomography; NGS, next-generation sequencing. ∗Only parameters with concordance rates of ≥50% with both the 90-gene assay and pathology/IHC are considered valuable clues.

This study included a total of 130 patients (Figure 1), 80 patients were from the SST group, and 50 were from the EC group. Baseline clinical characteristics were generally similar between the two groups (Table 1). Of the 130 patients, 51 (39%) were over the age of 60, and 79 (61%) were 60 years old or younger. 75 (58%) patients were male, and 55 (42%) were female, and all patients in this study were Chinese of Han ethnicity. Nearly all patients (124 [95%]) had an ECOG performance status of 0 or 1. In addition, 60 (46%) patients were pathologically diagnosed as adenocarcinoma, 41 (32%) as poorly differentiated carcinoma, and 29 (22%) as squamous cell carcinoma. Moreover, 35 (26.9%) patients had visceral metastasis, and 57 (43.8%) patients had bone metastasis. Furthermore, there were no significant differences in baseline clinical characteristics between the 130 patients and the intention-to-treat (ITT) population (Table S1).

**Table 1:** Baseline characteristics of 130 patients

Characteristic	Site-specific therapy (*n* = 80)	Empirical chemotherapy (*n* = 50)
Age (years)		
≤60	49(61.3%)	30(60.0%)
>60	31(38.7%)	20(40.0%)
Sex		
Male	46(57.5%)	29(58.0%)
Female	34(42.5%)	21(42.0%)
Race and Ethnicity		
Chinese of Han ethnicity	80(100.0%)	50(100.0%)
ECOG		
0-1	76(95.0%)	48(96.0%)
2	4(5.0%)	2(4.0%)
Histology		
Adenocarcinoma	40(50.0%)	20(40.0%)
Poorly differentiated carcinoma	22(27.5%)	19(38.0%)
Squamous cell carcinoma	18(22.5%)	11(22.0%)
Metastatic sites		
Visceral metastasis	21(26.3%)	14(28.0%)
Bone metastasis	37(46.2%)	20(40.0%)

### SST associated with PFS benefit

Of the 130 patients, the median PFS for the SST group was 9.5 months, compared to 5.6 months for the EC group, which had significant statistical and clinical significance (Figure S1A, 9.5 vs. 5.6 months, *p* = 0.025). The median OS of the SST group was 7.3 months longer, suggesting a clinically meaningful prolongation, though it was not statistically significant between the two groups (Figure S1B, 27.1 vs. 19.8 months, *p* = 0.184).

### The 90-gene assay and pathology/IHC

Between PPSs indicated by pathology/IHC and similarity scores with the 90-gene assay, there was no statistically significant difference, suggesting that a 90-gene assay score of ≤45% was also valuable for the diagnosis of PPS (Table S2).

### Other multimodal data with the 90-gene assay and pathology/IHC

Of the 130 patients, 64 patients had clues in pathology plus IHC that were suggestive of a single organ or body system, and 50 (78.1%) of whom were concordant with the 90-gene assay (Methods S1).

**Figure 2:**
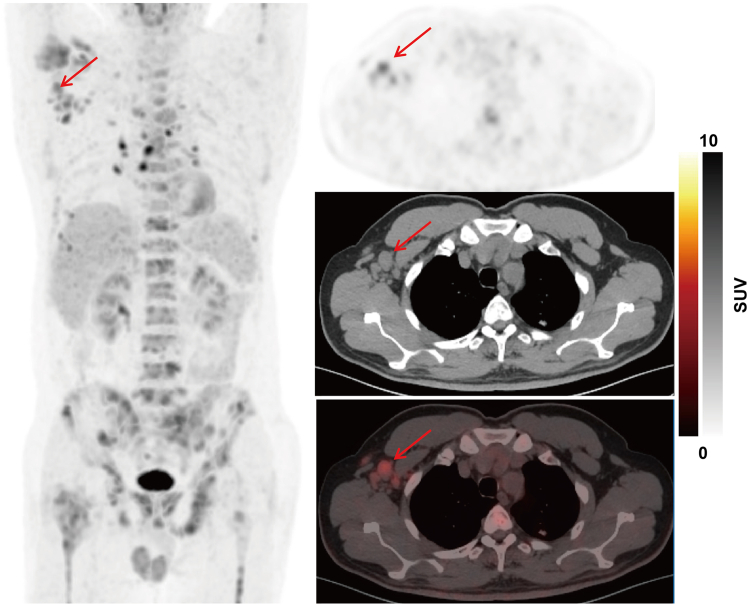
The putative primary in one CUP patient was predicted by the sentinel lymph node theory A 57-year-old man presented with pain in his right lower leg for six months. Physical examination was otherwise unremarkable. PET-CT scan demonstrated elevated ^18^F-FDG uptake in multiple bones and the right axillary lymph nodes. Biopsy of the right axillary lymph nodes revealed metastatic poorly-differentiated adenocarcinoma. IHC stains were negative for GATA3, CK20, P63, TTF1, Hep1, and ER, but positive for GCDFP15, AR, mammaglobin, and CK7. HER2 was 2+ but FISH negative. The pathology and IHC results suggested the primary tumor site was possibly the breast or accessory mammary gland. The 90-gene assay showed a similarity score of 79.3% for breast cancer. Breast MRI was unremarkable. Based on results from the pathology plus IHC and sentinel nodal theory (the right axillary lymph nodes), and taking into account the outcomes of the 90-gene assay, the MDT adjudicated that the diagnosis of this patient was a CUP/breast cancer staged as T0N1M1. The patient who had been randomized to the SST arm received albumin-bound paclitaxel plus cisplatin therapy with a PFS of 10.5 months and an OS of 23.8 months.

Among sentinel lymph nodes suggesting the primary sites, 15/22 (68.2%) and 4/6 (66.7%) were concordant with the 90-gene assay and pathology/IHC, respectively. It is noteworthy that among the 130 patients, 22 had sentinel lymph nodes. Of the 64 patients with indicative pathology/IHC findings, 6 had sentinel lymph nodes among them. Figure 2 showed that the PPS in one CUP patient was predicted by the sentinel lymph node theory. All FDG PET-CT images of sentinel lymph nodes in this study are shown in Appendix S1.

**Figure 3:**
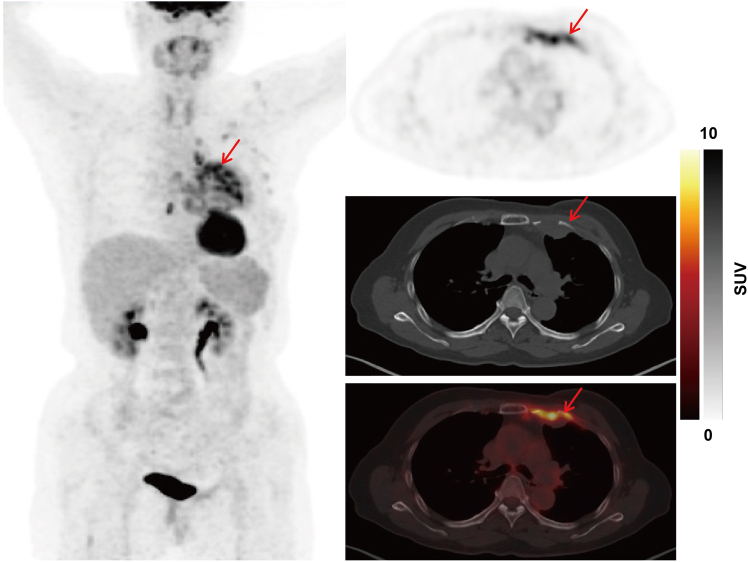
The putative primary site in one CUP patient was predicted by the Batson plexus theory A 59-year-old woman presented with a chest wall mass for two months. Physical examination was otherwise unremarkable. PET-CT scan demonstrated elevated ^18^F-FDG uptake in the left chest wall, the sternum, the second and third ribs. Post-operative pathology of the left chest wall mass revealed poorly-differentiated carcinoma. IHC stains were negative for ER, HER-2, mamaglobin, CK20, CDX-2, PAX8, WT1, Calretinin and D2-40, but positive for AE1/AE3, CK5/6, CK7, SMARCA4 BRG1, SMARCB1 (INI-1) and Ki-67(80%+). Pathology and IHC results were incapable of precisely determining the site of origin. The 90-gene assay indicated a similarity score of 52.3% for breast cancer. Based on results from Batson plexus (the second and third ribs) and the 90-gene assay, the MDT adjudicated that the diagnosis of this patient was a CUP/breast cancer subset staged as T0N0M1. The patient who had been randomized to the EC arm was treated with paclitaxel plus cisplatin with a PFS of 27.6 months and an OS of 27.6+ months.

In total, eight patients with solitary or limited bone metastasis in the torso had been adjudicated to have clues for PPSs based on Batson plexus theory; the concordant rate with the 90-gene prediction was 50% (4/8), while it was 75% (3/4) with pathology/IHC. Figure 3 showed that the PPS in one CUP patient was predicted by the Batson plexus theory. Other FDG PET-CT images of the Batson plexus in this study are shown in Appendix S2.

Serum tumor markers indicated the primary sites in 14 patients, with all markers indicating origin of digestive tract, and we did not observe abnormal elevations in alpha-fetoprotein (AFP) or prostate-specific antigen (PSA). The concordant rate with the 90-gene prediction was 42.9% (6/14) while it was 57.1% (4/7) with pathology/IHC.

Of the 39 patients with available NGS results, four harbored the described mutations. Specifically, these include one patient with anaplastic lymphoma kinase (ALK) mutation, one patient with ALK and ROS proto-oncogene 1 (ROS1) mutations, and two patients with epidermal growth factor receptor (EGFR) mutation. The concordant rate of NGS with the 90-gene prediction was 50.0% (2/4) while it was 66.7% (2/3) with pathology/IHC. However, we did not observe any clinical signs or symptoms suggestive of the PPSs in patients with CUP.

### PPS diagnosis by the 90-gene assay and the MDT

**Figure 4:**
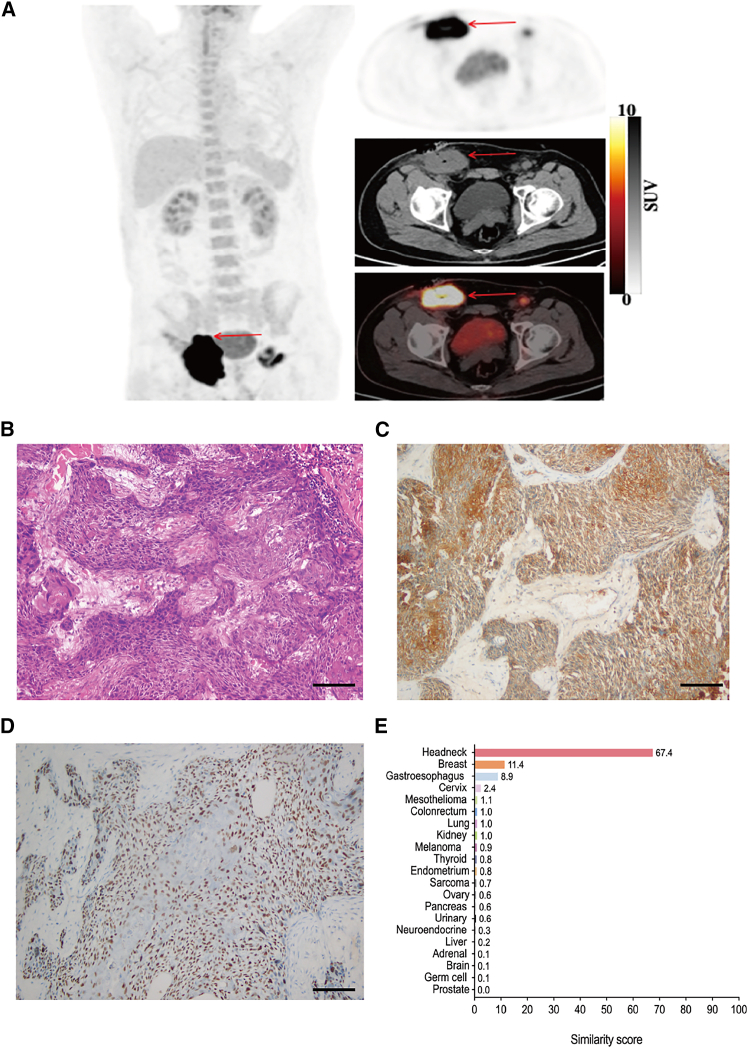
MDT in the management of CUP: diagnosis of presumptive primary tumors, TNM staging, and therapeutic strategies A 40-year-old man presented with a continuously enlarged subcutaneous mass in the right groin for nine months. Physical examination was otherwise unremarkable. (A) PET-CT scan demonstrated elevated ^18^F-FDG uptake within the mass in the right inguinal region and lymph nodes in the left inguinal region (arrow). (B) Biopsy of the right inguinal mass showed squamous cell carcinoma. Scale bars, 200 μm. (C and D) IHC stains were negative for EBER, TTF1, PAX8, GATA3, CK20, P16, and CK7, but positive for AE1/AE3 (C) and P63 (D). The pathology and IHC results suggested squamous differentiation, but could not indicate the site of origin. Scale bars, 200 μm. (E) The 90-gene assay showed a similarity score of 67.4% for head and neck squamous cell carcinoma. Based on results from the pathology and sentinel nodal theory (the right lymph nodes in the groin, red arrow), the MDT adjudicated that the diagnosis of this patient was a CUP/squamous cell carcinoma of the perineal region or right leg staged as T0N2M0. The patient who had been randomized to the EC arm received gemcitabine plus cisplatin therapy with a PFS of nine months and an OS of 22.6 months.

Regarding the diagnosis of putative primary cancers by the MDT, 85.4% (111/130) of patients showed PPS concordance with the 90-gene assay. Of note, the MDT revised the diagnoses based on the 90-gene assay predictions in five patients with CUP. Specifically, the diagnoses of four patients were revised from head and neck squamous cell carcinoma to squamous cell carcinoma of the perineal region or leg, while the diagnosis of the remaining one was revised from gastroesophageal carcinoma to lung or esophageal carcinoma (Figure 4, the remaining four cases in Appendix S3).

### Staging of CUP

Among the 130 patients, 127 (97.7%) patients were staged based on the 90-gene results, while 113 (86.9%) patients were successfully staged based on the MDT-adjudicated results. Among these 17 patients who were not staged, three patients could not be successfully staged due to their putative gastroesophageal origin. The remaining 14 patients were not staged because the PPS could not be assigned according to the MDT assessment.

### Prognostic value of clinical TNM staging

**Figure 5:**
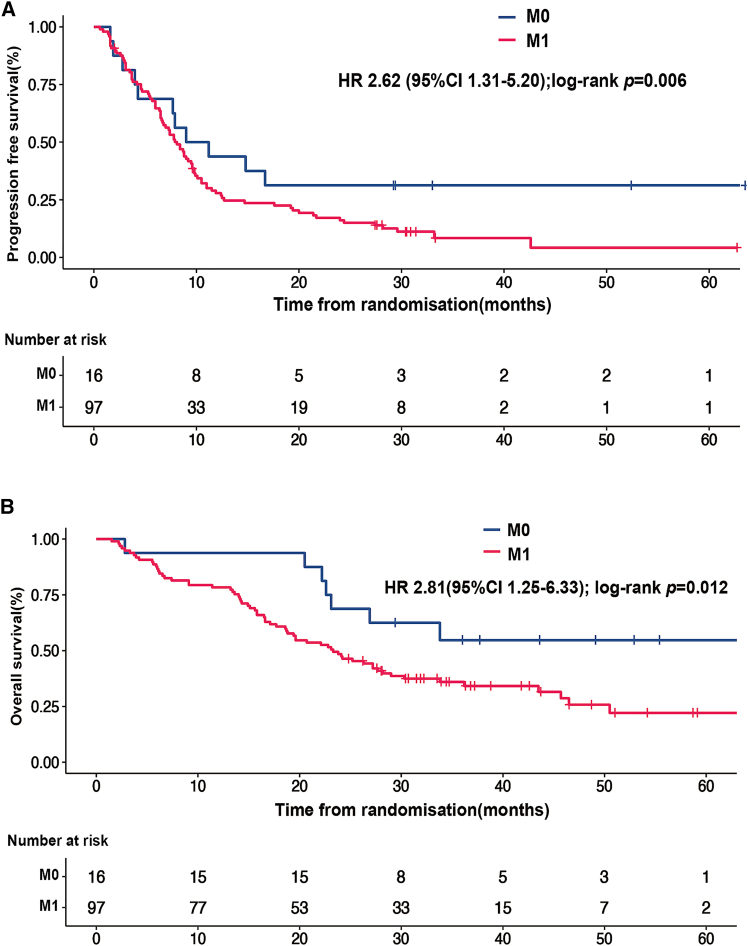
Kaplan-Meier survival plots between M0 and M1 stages based on the MDT-adjudicated results Kaplan-Meier plots for PFS (A) and OS (B) of patients with CUP between M0 (*n* = 16) and M1 (*n* = 97) stages based on the MDT-adjudicated results.

Based on the MDT-adjudicated findings, patients at the M0 stage presented significantly longer PFS in comparison to those at the M1 stage (Figure 5A, *p* = 0.006). For OS, M0 patients manifested a more favorable prognosis, with a significantly extended OS compared to M1 patients (Figure 5B, *p* = 0.012). Multivariate analyses showed that M1 stage and sex were two independent prognostic factors for PFS and OS (Tables S3 an S4). Based on the 90-gene assay results, patients at the M0 stage demonstrated a significantly longer PFS (Figure S2A, *p* = 0.043), but not for OS (Figure S2B, *p* = 0.151), compared to those at the M1 stage. Multivariate analyses showed that M1 stage and sex were two independent prognostic factors for PFS, while sex was the only independent factor influencing OS (Tables S5 and S6).

## Discussion

The randomized Fudan CUP-001 trial demonstrated that SST guided by the 90-gene assay significantly improved therapeutic outcomes, which was most likely to be driven by the use of targeted therapy and/or immunotherapy.^5^ For the diagnosis of PPS, GEP of tissue of origin, pathology/IHC, sentinel node, Batson plexus, and NGS all provided valuable clues, and the MDT may help to resolve the conflicting results. The high screen failure in the CUPISCO trial might reflect the complexity of CUP diagnosis.^24^ One strength of this study was that the diagnosis of putative primaries and clinical staging of CUP were adjudicated by the MDT comprising experienced experts of different specialties, based on the 90-gene results as well as their combination with all clinicopathologic features plus NGS results (if available).

Prior research suggests that 30% of patients with CUP can have their PPSs assigned through conventional pathology and IHC, which is based on epigenetic alterations.^17^^,^^25^ However, our study was able to assign PPS in 49.2% of cases. Moreover, its concordance rate with the 90-gene assay was 78.1%, which was consistent with the previously reported 71.6%.^8^ The reasons for discrepancy between sentinel nodes and the 90-gene assay may include positive sentinel nodes diagnosed by the MDT adjudication of PET-CT appearances, not by pathology, and rare cancer types not covered in the 90-gene assay, as well as gene expression overlaps (Figure 4).

The diagnosis of real clinical CUP requires a comprehensive analysis of the clinical and pathologic findings, as well as the 90-gene assay by the dedicated MDT team, which provides a platform for easy communications for any decision-making for diagnosis and treatment, and can sometimes see almost invisible clues for the diagnosis of primaries. The incidence of CUP has been declining mostly because of improved diagnostic technology to localize anatomical primaries. Imaging techniques, including CT, magnetic resonance imaging (MRI), and PET-CT scans, are typically used for primary site detection in clinics.^23^^,^^26^^,^^27^ Recently, the emergence of fibroblast activation protein inhibitor (FAPI) PET-CT has been shown to identify more primary sites, which made them excluded from the diagnosis of CUP.^28^^,^^29^ Like in this case, a primary site in the jejunum was identified using ^68^Ga-FAPI PET-CT, although no findings for all other tests, including FDG PET-CT and endoscopy (Figure S3). Therefore, it is highly likely that CUP is not a single biologically distinctive cancer, but rather a constellation of different cancer types with clinically undetectable anatomic primary sites at diagnosis.

There has been no staging system for all CUP, and its establishment will provide helpful information for both patients and physicians, assist in evaluating treatment results, and investigate treatment approaches. Based on our knowledge, this was the first effort to stage all patients with CUP, including both favorable and unfavorable patients with CUP, with 86.9% of patients with CUP being TNM stageable as per the MDT diagnosis in this study. Moreover, the M1 stage was associated with both poor PFS and OS, and was an independent prognostic factor for both of them.

It should be noted that GEP, such as the 90-gene assay, has to be used at the forefront in the diagnostic process of CUP.^24^^,^^30^^,^^31^ Other novel techniques, including NGS for in-depth genetic characterization, DNA methylation, and microRNA analyses to pinpoint tissue-specific patterns,^32^^,^^33^ and machine learning methods, have shown promise in the management of patients with CUP, warranting further clinical validations.

In conclusion, unlike the unpredictability of CUP metastasis reported by Olivier et al.,^34^ our data demonstrated that a minority of CUPs metastasize via the lymphatic and Batson plexus pathways, which can be exploited in FDG PET-CT imaging to provide important clues in the diagnosis of PPS. While the 90-gene assay is pivotal in the diagnosis of putative primary of CUP, its integration with other multimodal data could provide more reassuring evidence for the diagnosis of PPS. The majority of patients with CUP were TNM stageable based on the 90-gene assay and MDT. Designation of M1 stage by the MDT was an independent prognostic factor for both PFS and OS. This study addressed the role of the tissue of origin-related GEP, pathology, plus IHC, NGS, FDG PET-CT, and MDT in the context of clinical features in the management of CUP.

### Limitations of the study

Limitations of this study should be acknowledged. First, five patients with CUP with the MDT revisions of PPS diagnosis did not receive corresponding first-line medical treatment, since the Fudan CUP-001 trial was a randomized trial and patients had to receive either the 90-gene assay-directed SST or EC as per protocol. Second, the MDT process was derived from a single specialized center, which may limit its generalizability. We are now actively evaluating it in multiple hospitals at different levels. Third, the unavailability of NGS results of all patients may overestimate the overall clinical utility in an unselected population, while this reflects performance among patients with detectable alterations. Fourth, the survival comparison between the SST and EC groups within the 130-patient cohort was a post-hoc, non-randomized analysis with potential for group imbalance. Fifth, confirmation bias could be present with any participating expert as to any parameter (including the 90-gene assay).

## Resource availability

### Lead contact

Further information and requests for resources should be directed to and will be fulfilled by the Lead Contact, Xichun Hu (huxichun2017@163.com).

### Materials availability

This study did not generate new unique reagents.

### Data and code availability

• Data: This paper analyzes existing, publicly available data, accessible at DOI: https://doi.org/10.1016/S1470-2045(24)00313-9. All data reported in this paper will be shared by the lead contact upon request.
• Code: This study did not report original codes.
• Any additional information required to reanalyze the data reported in this paper is available from the lead contact upon request.

## Acknowledgments

This study was supported by the 10.13039/501100001809National Natural Science Foundation of China (82473071; 82430092), the 10.13039/501100018537National Science and Technology Major Project (2025ZD0545500, 2025ZD0545501), Program for Shanghai Outstanding Academic Leader (LJRC2102), and 10.13039/100017633Shanghai Anticancer Association SOAR PROJECT (SACA-AX202402).

## Author contributions

The study was designed by X.H., Q.W., H.W., S.H., Z.L., and S.P. S.P. and X.H. wrote the manuscript. Q.W., H.W., S.H., Z.L., J.W., J.L., and S.H. conducted the literature search and revised the manuscript. The pathological data were collected and adjudicated by J.L., Q.W., Y.W., and X.Z.; the imaging data by H.L. and L.Z.; the PET-CT imaging data by S.H., S.H., and S.S.; the clinical data by X.L., X.Z., S.J., and all other clinical experts. The data analysis was carried out by S.P. and J.L. under the supervision of X.H. X.H., Q.W., H.W., S.Hu, and Z.L. co-supervised the study. All authors had full access to all the data in the study and had final responsibility for the decision to submit for publication.

## Declaration of interests

The authors have declared no conflicts of interest.

## STAR★Methods

### Key resources table

**Table:** 

REAGENT or RESOURCE	SOURCE	IDENTIFIER
**Biological samples**		

CUP tumor tissue samples	Fudan university shanghai cancer center	N/A

**Critical commercial assays**		

90-gene assay	Canhelp Genomics Co., Ltd	N/A

**Deposited data**		

Analyzed data	Liu et al.^5^	https://doi.org/10.1016/S1470-2045(24)00313-9

**Software and algorithms**		

R software (version 4.4.2)	the R Core Team and the R Foundation for Statistical Computing	https://www.r-project.org/
SPSS (Version 22)	IBM Corp	https://www.ibm.com/spss

### Experimental model and study participants details

#### Patients

The Fudan CUP-001 trial was a randomised controlled trial conducted at Fudan University Shanghai Cancer Center (FUSCC; Shanghai, China).^5^ Patients were randomly allocated (1:1) to either empirical treatment or site-specific treatment. For site-specific therapy, an archived FFPE tumor specimen was analyzed in an approved clinical laboratory using the Canhelp-Origin 90-gene expression assay. For the empirical chemotherapy group, patients were given taxane (175 mg/m^2^ by intravenous infusion on day 1) plus platinum (cisplatin 75 mg/m^2^ or carboplatin area under the curve 5 by intravenous infusion on day 1), or gemcitabine (1000 mg/m^2^ by intravenous infusion on days 1 and 8) plus platinum (same as above), per physician’s choice, administered every 3 weeks until disease progression, death, unacceptable adverse events, withdrawal of informed consent, or a maximum of six cycles. The primary endpoint was PFS, defined as the interval from the time of randomisation to disease progression or death from any cause, whichever occurred first. Secondary endpoints were OS, objective response rate, safety, and biomarker investigation.

A total of 130 patients were included in this study. The baseline characteristics, including age, sex, race, ethnicity, and ECOG performance status, are presented in Table 1. The 90-gene assay was successfully performed on these patients, with 80 patients in the experimental arm as per protocol and 50 patients in the control arm which had been required by the peer reviewers. All patients had undergone a standard evaluation, including medical history, physical examination, blood counts, chemistry profile, serum tumor marker patterns, FDG PET-CT scans, endoscopic examination when clinically indicated, and pathologic examination. The baseline clinical characteristics of the 130 patients and the ITT population were compared.

#### Ethics

The protocol and all amendments were approved by the institutional ethics review board at FUSCC (1707174-12-2103B). The trial was performed according to the principles of the Declaration of Helsinki and Good Clinical Practice guidelines. Written informed consent was obtained from all the patients before enrollment.

### Method details

#### Similarity score of the 90-gene assay with pathology/IHC

Based on the previously established cutoff similarity score of 45% for the 90-gene assay,^8^ 130 patients were divided into two groups, 84 patients with scores >45% and 46 patients with scores ≤45%. Based on the concordance status between the 90-gene assay and pathology/IHC for prediction of PPS of CUP, they were categorized for assessment of the association between similarity scores of the 90-gene assay and pathology plus IHC.

#### Comparisons between multimodal parameters

Assessments of tissue of origin presumptions on routine pathology and IHC had been done by two independent pathologists who are specialized in pathologic diagnosis of CUP to determine whether they were suggestive of one organ or one body system, and there would be a thorough discussion if the results were discordant. The IHC antibodies for CUP, as summarized in Table S7, were applied during the second or later round of testing.

Four clinical parameters (characteristic symptoms, sentinel lymph nodes, vertebral venous plexus [Batson plexus], tumor marker patterns) and NGS were chosen on the basis of prior reports.^19^^,^^20^ Positive sentinel nodes which had been adjudicated to be clues were determined based on the size, limitation to the locoregional area, distribution, and FDG uptake of lymph nodes. The bones in the torso are divided into the three parts, that is, the cervical bone, chest bone (sternum, rib and thoracic vertebra) and lumbar/pelvis bone. CUP patients with bone metastasis limited to one of the three parts were judged to have clues, after exclusion of patients with simultaneous visceral metastasis which might suggest possibility of systemic spreading.

Tumor biomarkers included AFP for liver cancer, beta-human chorionic gonadotropin (β-hCG) for tumors of the reproductive system, PSA for prostate cancer; Elevations of at least 2-fold in three or more biomarkers among CA19-9, CA72-4, CA50, and CA242 were considered indicative of gastrointestinal origin.^23^^,^^35^^,^^36^

Suggestive NGS clues included ALK, ROS1 and EGFR for non-small cell lung cancer (NSCLC), transmembrane serine protease 2 (TMPRSS2) for prostate cancer, and rearrangements of nuclear protein in testis midline carcinoma family member 1 (NUTM1) for NUT carcinoma.^23^

#### MDT

A specialized MDT for CUP in our center has been holding meetings weekly since 2017. This specialized MDT required at least one expert that have at least 10 years of experience in diagnosis and differential diagnosis of cancer types for all specialties (medical oncology, pathology, diagnostic radiology, and diagnostic nuclear medicine at least) to take part in MDT meeting.

The MDT process starts with case history presentation, determination of a diagnosis of cancer of unknown primary and any clue in clinical manifestation and serum tumor markers as well as NGS by the team, determination of any clue for sentinel lymph node and Batson’s plexus in PET-CT images by nuclear medicine specialists and other diagnostic imaging (e.g., CT, MRI) by radiologists, and then pathologist interpretation of pathology/IHC results and the 90-gene assay results. The diagnoses of PPSs were adjudicated following the protocol (Methods S1 and Figure 1). A presumptive primary site was suggested and documented, which would not impact on patient’s treatment on the Fudan CUP-001 trial since site specific therapy was guided by the 90-gene assay alone rather than the results of the MDT.

After the public releasing of CUP-001 results on July 25, 2024, we wanted to publish our diagnostic experience. A valuable clue for PPS diagnosis was set and should meet the criteria that have ≥50% concordance rate with both 90-gene assay (validated in the prospective clinical trial) and Pathology/IHC suggestions (well recognized in the clinic). The clinical feature and serum tumor marker patterns were discarded because of not meeting the criteria and the PPS results were the exactly same as those documented in the case records. Clinical staging was designated based on the 90-gene and MDT results following the eighth edition American Joint Committee on Cancer (AJCC) Cancer Staging Manual for corresponding primary-known cancers.^37^^,^^38^

### Quantification and statistical analysis

This was a post-hoc analysis of the Fudan CUP-001 trial to explore the diagnostic clues for PPSs. Due to the paucity of published data in this specific research domain, formal sample size calculation was not performed for this exploratory study.

In this study, PFS and OS outcomes were compared between the SST group and the EC groups in the 130 patients. To explore the performance of identified prognostic factors (age, sex, pathology, treatment regimen, and clinical TNM stages based on the 90-gene assay or MDT results) on progression-free and overall survival, a post-hoc analysis using unstratified Cox proportional hazard models was done.

PFS and OS were estimated using Kaplan–Meier method and compared using the log rank test. Relations between the 90-gene assay and pathology plus IHC were analyzed with Chi-square test. SPSS (Version 22) and R 4.4.2 were used for statistical analysis. All statistical tests were with the significant level being set at *p* < 0.05.

### Additional resources

This study is derived from the Fudan CUP-001 randomized trial (NCT03278600).
