# Supplementary material for: Multimodal parameters for the diagnosis of putative primaries of cancer of unknown primary
Source: iScience. 2026 Apr 1;29(5):115558. doi: 10.1016/j.isci.2026.115558 (PMC13091557; doi:10.1016/j.isci.2026.115558)
Supplement: Document S1. Figures S1–S3, and Tables S1–S7, Methods S1, and Appendix S1–S3 [file mmc1.pdf]

iScience, Volume *29*

Supplemental information

Multimodal parameters for the diagnosis

# of putative primaries of cancer of unknown primary

Shuangyue Pan, Jinyan Wang, Jing Liu, Simin He, Xin Liu, Xiaowei Zhang, Shiyu Jiang, Yanli Wang, Xiaoyan Zhou, Shaoli Song, Liangping Zhou, Haiming Li, Jianfeng Luo, Zhiguo Luo, Silong Hu, Hongxia Wang, Qifeng Wang, and Xichun Hu

Supplemental figures

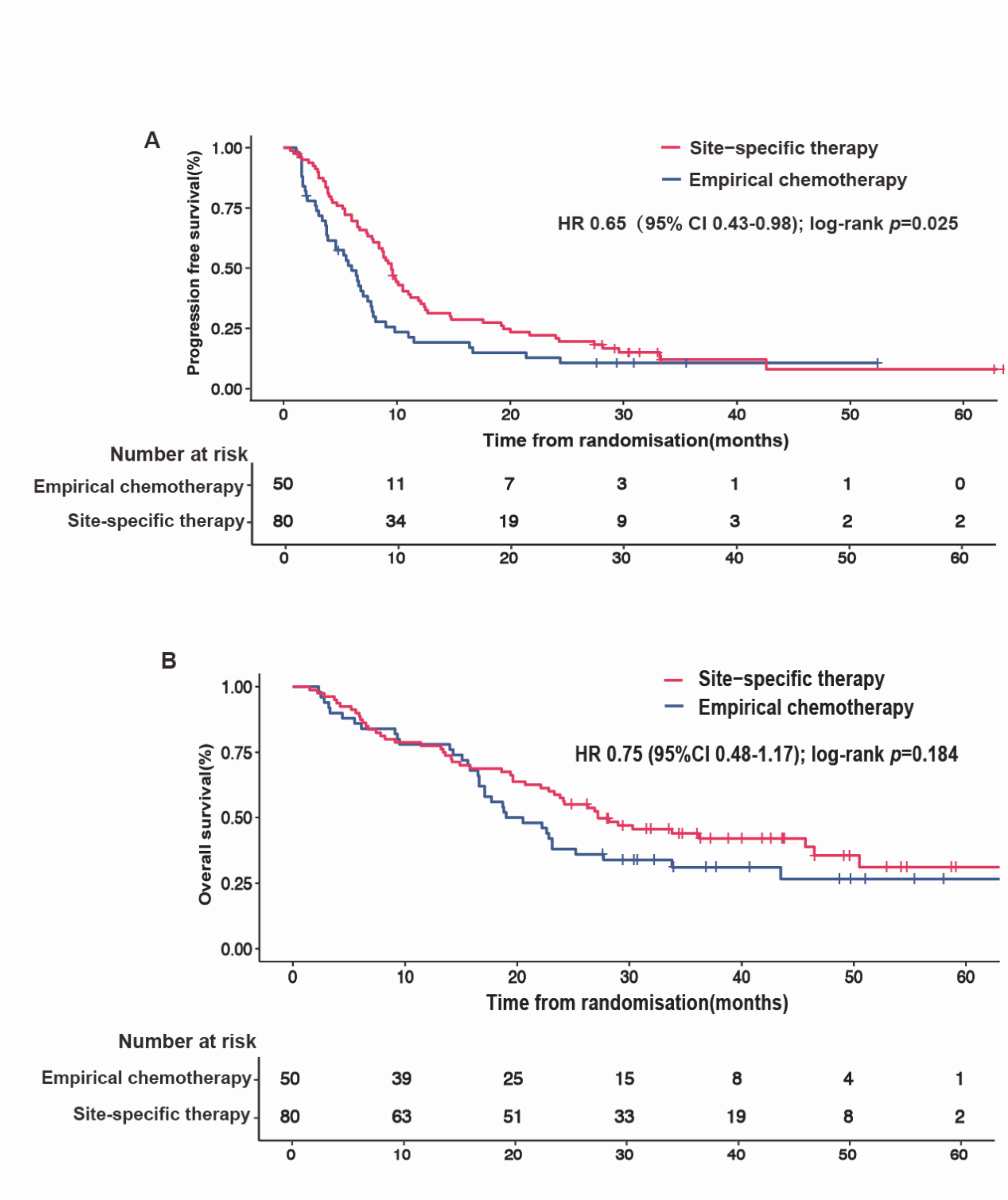

Figure S1. Kaplan–Meier survival plots in the 130 patients. Kaplan–Meier plots for PFS (A) and OS (B) according to site-specific (n=80) or empirical therapy (n=50) in the 130 patients with the 90-gene assay.

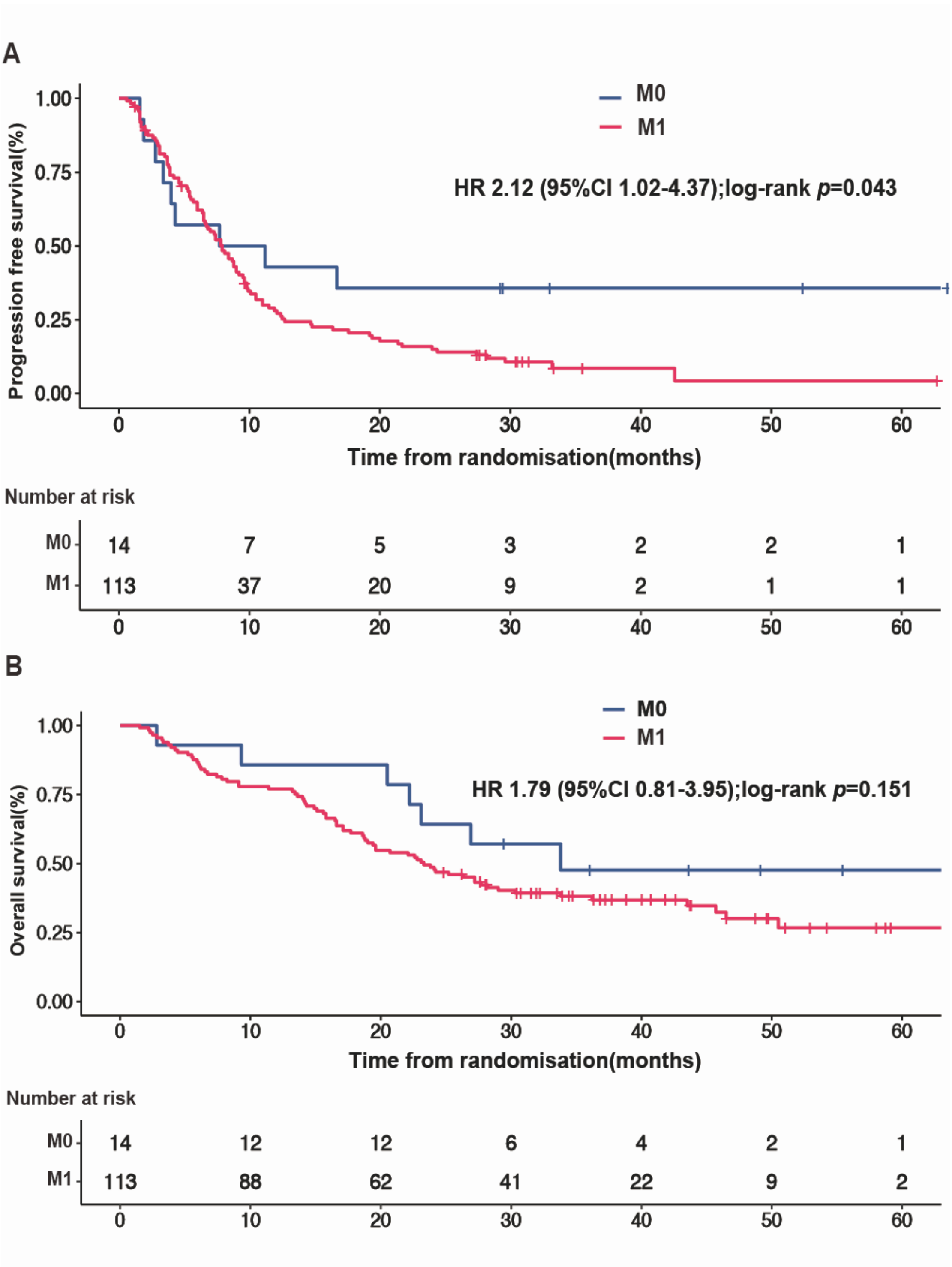

Figure S2. **Kaplan–Meier survival plots between M0 and M1 in the patients based on the 90-gene assay.** Kaplan–Meier plots for PFS (**A**) and OS (**B**) according to M0 (n=14) and M1 (n=113) in the patients based on the 90-gene assay.

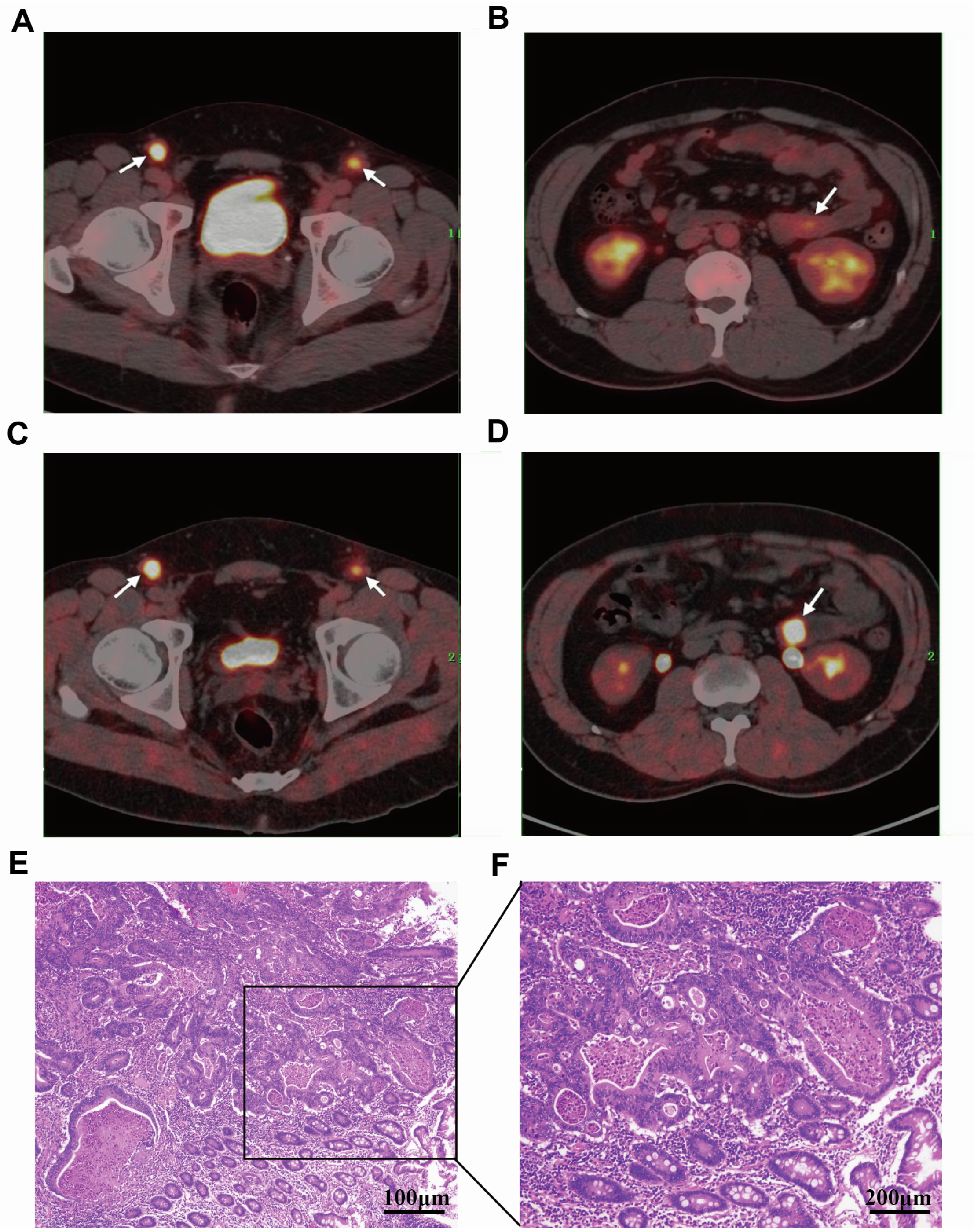

Figure S3. ^{68}Ga FAPI PET/CT, not FDG PET-CT identified a primary lesion in the jejunum, leading to the total resection of all involved lesions and adjuvant treatment. A 39-year-old nonsmoking man with bilateral inguinal lymph node enlargement and CEA increase (58.53ng/ml). Biopsy revealed poorly differentiated carcinoma, possibly adenocarcinoma. FDG PET-CT scan showed multiple hypermetabolic nodes in the inguina (**A**, arrow) and no definite evidence of primary cancer (**B**, arrow). ^{68}Ga FAPI PET/CT showed the same enlarged lymph nodes (**C**, arrow) and primary cancer in the jejunum (**D**, arrow). Postoperative pathology revealed a differentiated adenocarcinoma in the jejunum, infiltrating into the serous membrane, with 6/18 positive nodes (**E-F**). Up to October 27, 2024, the patient has been disease-free for 10 months in terms of serum tumor marker, ctDNA and conventional abdominal/ pelvis CT tests. Scale bars: 100μm (10×) and 200μm (20×).

Supplemental tables

Table S1. Comparison of baseline characteristics between 130 patients and ITT population

Characteristic	ITT population (n=182)	Patients with 90-gene assay (n=130)	*p* value[a]
**Age(years)**			
≤60	106 (58.2%)	79 (60.8%)	0.726
>60	76 (41.8%)	51 (39.2%)	
**Sex**			
Male	105 (57.7%)	75 (57.7%)	>0.999
Female	77 (42.3%)	55 (42.3%)	
**Ethnicity**			
Asian	182 (100%)	130 (100%)	/
**ECOG**			
0-1	174 (95.6%)	124 (95.4%)	
2	8 (4.4%)	6 (4.6%)	>0.999
**Histology**			
Adenocarcinoma	84 (46.2%)	60 (46.2%)	
Poorly differentiated carcinoma	57 (31.3%)	41 (31.5%)	0.869
Squamous cell carcinoma	40 (22.0%)	29 (22.3%)	
Poorly differentiated neoplasm	1 (0.5%)	0	
**Metastatic sites**			
Visceral metastasis	53 (29.1%)	35 (26.9%)	0.670
Bone metastasis	77 (42.3%)	57 (43.8%)	0.790

[a]Differences between ITT population and patients with the 90-gene assay were compared using the Pearson's chi-square test.

Table S2. Similarity score of the 90-gene assay with pathology/IHC

	Pathology plus IHC			p value[a]
	Concordant with 90-gene assay	Discordant with 90-gene assay	Not suggestive[b]	
≤ 45%[c] n=46	16	8	22	0.195
> 45% n=84	34	6	44	

[a] p value was calculated using the Pearson's chi-square test.

[b] Among 130 patients, pathology/IHC results suggested one organ or one body system in 64 cases, while the remaining 66 showed non-suggestive findings.

[c] The cutoff similarity score of 45% for the 90-gene assay.

Table S3. Prognostic factors for PFS in CUP

	HR (95% CI)	*p* value[a]
Site-specific therapy vs Empirical chemotherapy	0.72 (0.43-1.21)	0.217
Targeted therapy and/or immunotherapy vs no targeted therapy or immunotherapy	0.68 (0.40-1.14)	0.143
M1 vs M0[b]	2.62 (1.31-5.20)	0.006
Age	0.99 (0.97-1.01)	0.150
Sex	0.41 (0.26-0.65)	<0.001
Pathology		0.659
Poorly differentiated carcinoma vs Adenocarcinoma	0.87 (0.53-1.43)	0.594
Squamous carcinoma vs Adenocarcinoma	0.77 (0.44-1.36)	0.364

Notes:

[a]*p* values were calculated using unstratified Cox proportional hazards regression models.

[b]The TNM staging of these patients was based on the MDT-adjudicated results.

Table S4. Prognostic factors for OS in CUP

	HR (95% CI)	*p* value[a]
Site-specific therapy vs Empirical chemotherapy	0.84 (0.47-1.49)	0.541
Targeted therapy and/or immunotherapy vs no targeted therapy or immunotherapy	0.74 (0.40-1.39)	0.349
M1 vs M0[b]	2.81 (1.25-6.33)	0.012
Age	0.99 (0.97-1.02)	0.454
Sex	0.47 (0.28-0.77)	0.003
Pathology		0.987
Poorly differentiated carcinoma vs Adenocarcinoma	0.97 (0.55-1.69)	0.900
Squamous carcinoma vs Adenocarcinoma	1.01 (0.53-1.93)	0.980

Notes:

[a]*p* values were calculated using unstratified Cox proportional hazards regression models.

[b]The TNM staging of these patients was based on the MDT-adjudicated results.

Table S5. Prognostic factors for PFS in CUP

	HR (95% CI)	*p* value[a]
Site-specific therapy vs Empirical chemotherapy	0.85 (0.54-1.35)	0.485
Targeted therapy and/or immunotherapy vs no targeted therapy or immunotherapy	0.64 (0.39-1.07)	0.087
M1 vs M0[b]	2.12 (1.02-4.37)	0.043
Age	0.99 (0.97-1.01)	0.130
Sex	0.51 (0.33-0.77)	0.002
Pathology		0.504
Poorly differentiated carcinoma vs Adenocarcinoma	0.83 (0.53-1.30)	0.417
Squamous carcinoma vs Adenocarcinoma	0.73 (0.42-1.26)	0.259

Notes:

[a]*p* values were calculated using unstratified Cox proportional hazards regression models.

[b]The TNM staging of these patients was based on the results of the 90-gene assay.

Table S6. Prognostic factors for OS in CUP

	HR (95% CI)	*p* value[a]
Site-specific therapy vs Empirical chemotherapy	0.84 (0.50-1.43)	0.518
Targeted therapy and/or immunotherapy vs no targeted therapy or immunotherapy	0.84 (0.46-1.53)	0.575
M1 vs M0[b]	1.79 (0.81-3.95)	0.151
Age	0.99 (0.97-1.01)	0.387
Sex	0.55 (0.34-0.87)	0.012
Pathology		0.947
Poorly differentiated carcinoma vs Adenocarcinoma	1.04 (0.62-1.73)	0.897
Squamous carcinoma vs Adenocarcinoma	1.11 (0.59-2.08)	0.743

Notes:

[a]*p* values were calculated using unstratified Cox proportional hazards regression models.

[b]The TNM staging of these patients was based on the results of the 90-gene assay.

Table S7. Immunohistochemistry antibodies in the Fudan CUP-001 trial

Tumor site or type	Cytokeratin 7 (CK7) and Cytokeratin 20 (CK20)	Primary markers[a]
Breast, sweat or salivary glands	CK7+/CK20-	GATA3±<br>AR+<br>GCDFP15±<br>Mammaglobin±
Lung adenocarcinoma	CK7+/CK20-	TTF-1+<br>Napsin A±
Renal cell carcinoma	CK7±/CK20-	PAX8+<br>Vimentin+<br>CD10±
Ovarian carcinoma	CK7+/CK20-	PAX8+<br>CA125+<br>WT1±
Cervical carcinoma	CK7-/CK20-	P40+<br>CK5/6+<br>P63+<br>P16+
Urothelial carcinoma	CK7+/CK20±	GATA3+<br>CK5/6+<br>P63+
Neuroendocrine carcinoma	CK7±/CK20-	Syn±<br>CgA±<br>CD56+<br>INSM1±
Mesothelioma	/	Calretinin+<br>WT1+<br>CK5/6+
Head and neck squamous cell carcinoma	/	P63+<br>P40+<br>EBER+
Digestive system[b]	CK7±/CK20±	CDX-2±、Villin±、SMAD4±、<br>CEA+、MUC1+、MUC5AC+

Female reproductive system[c]	CK7+/CK20-	PAX8+<br>WT1±<br>HNF1β+

Notes:

[a] Immunohistochemistry antibody selection was carried out according to the 2024 NCCN guidelines. The results of primary markers meet one or more to diagnose the corresponding tumor.

[b] Digestive system tumors include gastroesophageal cancer, colorectal cancer, pancreatic cancer, and hepatocellular carcinoma.

[c] Female reproductive system tumors include germ cell carcinoma and endometrial carcinoma.

Methods S1. CUP diagnosis protocol in CMUP center, FUSCC

CUP diagnosis protocol in CMUP center, FUSCC

Original protocol

1. Definition of clues for putative primary sites (PPS): the 90-gene assay, pathology/IHC, sentinel lymph nodes, Batson plexus, serum tumor marker patterns, sign and symptom, and NGS.

    a) The 90-gene assay: The 90-gene expression assay (Canhelp-Origin Test [Canhelp Genomics, Hangzhou, China]) is a real-time PCR-based assay that uses differential gene-expression patterns to assign putative tumor types in one of 21 tumor types in its spectrum[1]. It has been approved by the National Medical Products Administration (NMPA), China; and has passed the European Council Directive 98/79/EC. However, as reported, this gene expression profiling has an accuracy of 80% to 90%, while 10% to 20% of predicted putative primary sites are inaccurate mostly because of rare cancers and gene expression overlaps.

    b) Pathology/IHC: Pathology and IHC had been done by two independent pathologists and were discussed whether they were suggestive of one organ or one body system.

    c) Sentinel lymph nodes: 1) CUP patients with locoregional lymph node metastases restricted to 1-2 lymphatic drainage pathways; 2) exclusion criteria: supraclavicular node involvement only whose PPS cannot be deduced, or extensive non-locoregional lymph nodes, or a history of previous malignancy (except non-melanoma skin cancer or in situ carcinoma of the cervix) [2]. The judgment of presence of sentinel lymph node clue should follow the lymph node spreading pattern of the corresponding primary-known cancers, and all structural and metabolic information of lymph nodes throughout the whole body should be provided as in PET/CT.

    d) Batson plexus: Based on our prior study, the bones in the torso are divided into the three parts, that is, the cervical bone, chest bone and lumbar/pelvis bone.

CUP patients with bone metastasis limited to one of the three parts was judged to have clues, after exclusion of patients with simultaneous visceral metastasis which may suggest possibility of systemic spreading[3]. All structural and metabolic information of bones throughout the whole body should be provided as in PET/CT.

e) Serum tumor marker patterns:
   1) Alpha-fetoprotein (AFP) for liver cancer or hepatoid adenocarcinoma[4]
   2) Beta-human chorionic gonadotropin (β-hCG) for tumors of the reproductive system[5]
   3) Prostate-specific antigen (PSA) for prostate cancer[6]
   4) At least two times elevations in at least three of CA19-9, CA72-4, CA50, and CA242, were adjudicated to be indicative of the gastrointestinal origin[7-11]

f) Sign and symptom. Presence of any of the following:
   1) Progressive dysphagia for esophageal cancer[12]
   2) Retractable oral bleed or epistaxis for nasopharyngeal cancer[13]
   3) History or presence of Paget's disease[14,15]

g) Next-generation sequencing (NGS). Presence of any of the following:
   1) Anaplastic lymphoma kinase (ALK), ROS proto-oncogene 1 (ROS1) and epidermal growth factor receptor (EGFR) for NSCLC[16]
   2) Transmembrane serine protease 2 (TMPRSS2) for prostate cancer[17]
   3) Rearrangements of nuclear protein in testis midline carcinoma family member 1 (NUTM1) for NUT carcinoma[18]

2. PPS diagnostic procedure
   a) MDT judgment of presence or absence of any clues for PPS.
   b) A decision on PPS was made when no conflicting results.
   c) A consensus was reached when any conflicting results. PPS was adjudicated by the team.

Table. Value of multimodal parameters in deducing the PPS of CUP

	Sentinel lymph nodes	Batson plexus	Serum tumor marker	NGS	Sign and symptom
90-gene assay	15/22 (68.2%)	4/8 (50.0%)	6/14 (42.9%)	2/4 (50.0%)	0/0
Pathology/IHC	4/6 (66.7%)	3/4 (75.0%)	4/7 (57.1%)	2/3 (66.7%)	0/0

Note: for the assignment of tissue of origin of any cancer tissue, the 90-gene assay has been validated in a prospective randomized phase 3 trial, and pathology/IHC is the routine pathologic practice, therefore, all other parameters were compared with those two methods in patients with the two results available. x/y, x stands for number of cases with rank variable clue consistent with the row variable clue, y stands for number of cases with clues for both variables. ≥ 50% concordant rate of the parameter with both of the two methods was considered to be valuable in deducing the PPS.

Updated protocol after CUP-001

Based on the comparisons of sentinel lymph nodes, Batson plexus, serum tumor marker patterns, sign and symptom, NGS with the 90-gene assay which has been validated in the prospective randomized trial, and pathology/IHC which has been standard practice in routine pathology, the five parameters, that is, the 90-gene assay, pathology/IHC, sentinel lymph nodes, Batson plexus, and NGS highly suggestive of tissue of origin, was considered to be capable of providing diagnostic clues for PPS.

1. Definition of clues for putative primary sites (PPS). The establishment of a PPS only factors for the 90-gene assay, pathology/IHC, sentinel lymph nodes, Batson plexus, and NGS.
   a) The 90-gene assay: The 90-gene expression assay (Canhelp-Origin Test [Canhelp Genomics, Hangzhou, China]) is a real-time PCR-based assay that uses differential gene-expression patterns to assign putative tumor types in one of 21 tumor types in its spectrum[1]. It has been approved by the National Medical Products Administration (NMPA), China; and has passed the European Council Directive 98/79/EC. However, as reported, this gene expression profiling has an accuracy of 80% to 90%, while 10% to 20% of predicted putative primary sites are inaccurate mostly because of rare cancers and gene expression overlaps.
   b) Pathology/IHC: Pathology and IHC will be done by two independent pathologists and are discussed whether they are suggestive of one organ or one body system.
   c) Sentinel lymph nodes: 1) CUP patients with locoregional lymph node metastases restricted to 1-2 lymphatic drainage pathways; 2) exclusion criteria: supraclavicular node involvement only whose PPS cannot be deduced, or extensive non-locoregional lymph nodes, or a history of previous malignancy (except non-melanoma skin cancer or in situ carcinoma of the cervix) [2]. The judgment of presence of sentinel lymph node clue should follow the lymph

node spreading pattern of the corresponding primary-known cancers, and all structural and metabolic information of lymph nodes throughout the whole body should be provided as in PET/CT.

d) Batson plexus: Based on our prior study, the bones in the torso are divided into the three parts, that is, the cervical bone, chest bone and lumbar/pelvis bone. CUP patients with bone metastasis limited to one of the three parts are judged to have clues, after exclusion of patients with simultaneous visceral metastasis which may suggest possibility of systemic spreading[3]. All structural and metabolic information of bones throughout the whole body should be provided as in PET/CT.

e) Next-generation sequencing (NGS). Presence of any of the following:
   1) Anaplastic lymphoma kinase (ALK), ROS proto-oncogene 1 (ROS1) and epidermal growth factor receptor (EGFR) for NSCLC[16,19]
   2) Transmembrane serine protease 2 (TMPRSS2) for prostate cancer[17,19]
   3) Rearrangements of nuclear protein in testis midline carcinoma family member 1 (NUTM1) for NUT carcinoma[18,19]

2. PPS diagnostic procedure
   a) MDT judgment of presence or absence of any clues for PPS.
   b) A decision on PPS is made when no conflicting results. A consensus will be reached when any conflicting results. PPS is adjudicated by the team.
   c) If only the 90-gene result is available with no other clues, the PPS will be the one suggested by this assay.
   d) Diagnosis description, CUP/ XX cancer.
   e) TNM staging will be done accordingly. According to the putative primary sites designated by the MDT, we determine the M category in the clinical TNM staging of patients based on the AJCC guidelines as for primary-known cancers. Generally, patients with distant metastases are classified as M_1, while those without distant metastases are classified as M_0.

Appendix 1. Sentinel node theory

CASE 1

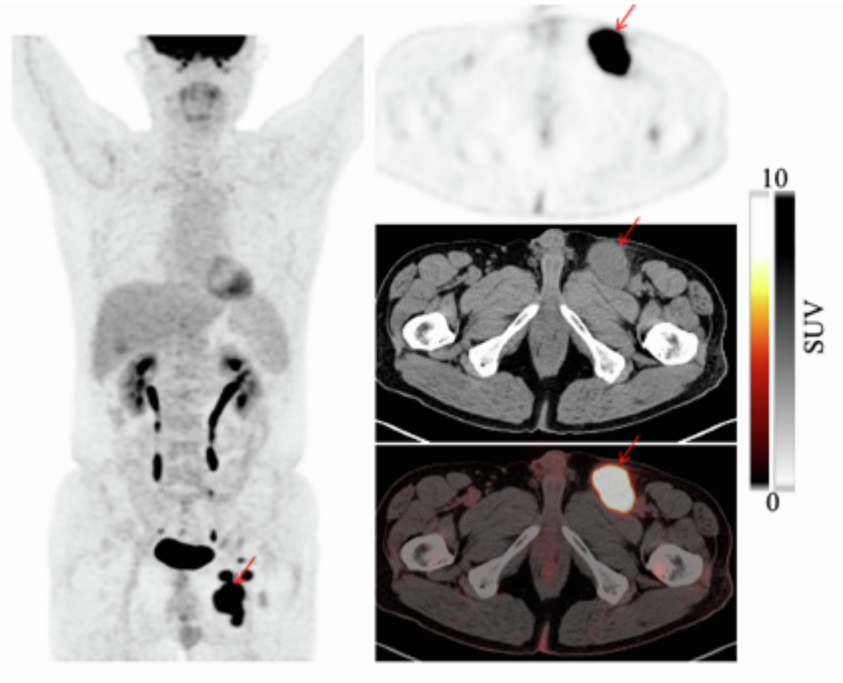

A 65-year-old man presented with continuously enlarged lymph nodes in the left groin for six months. Physical examination was otherwise unremarkable. PET-CT scan showed increased ^{18}F-FDG uptake in the left inguinal and paravascular iliac lymph nodes, being consistent with cancer metastasis. Biopsy of the left inguinal lymph node revealed focal squamous differentiation. IHC stains were negative for GATA3, SOX10, HMB45, SALL4, Oct4, PSA, CDX2, Syn, CgA, CD56, AR, Arg-1, GPC-3, and EBER, but positive for AE1/AE3, P40, INI-1, and SMACA4. The pathology and IHC results suggested squamous differentiation but could not indicate the site of origin. The 90-gene assay showed a similarity score of 43% for head and neck squamous cell carcinoma. NGS identified mutations in ARID1A, EPHB1, KMT2C, PARP1, PIK3C2G, SMAD4, and TP53. Based on results from the pathology plus IHC and sentinel nodal theory (the left inguinal lymph node), the MDT adjudicated that the diagnosis of this patient was a CUP/ squamous cell carcinoma of the perineal region or left leg staged as T0N1M0. The patient who had been randomized to the EC arm received gemcitabine plus cisplatin therapy with a PFS of 7.9 months and an OS of 37.7 months.

CASE 2

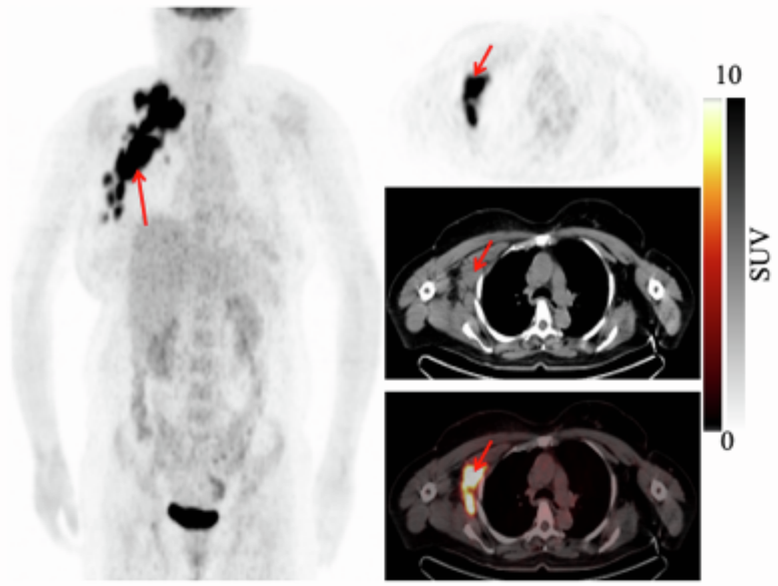

A 50-year-old woman presented with enlarged lymph nodes in the right lower neck. Physical examination was otherwise unremarkable. PET-CT scan demonstrated multiple enlarged lymph nodes with high ^{18}F-FDG uptake in the right lower neck, supraclavicular region, interpectoral space, and axilla. Biopsy of the lymph nodes in the right lower neck revealed a poorly-differentiated carcinoma, showing a predilection for adenocarcinoma. IHC stains were negative for ER, PR, mammaglobin, PAX8 and TTF-1, but positive for AE1/AE3, AR, CK7, GATA3, GCDFP15 and HER2, suggesting the putative primary lesion being the breast. The breast MRI did not indicate remarkable lesions in the breast. The 90-gene assay indicated a similarity score of 83.9% for breast cancer. In light of the results from the pathology plus IHC, the sentinel nodal theory (the right axillary lymph node), and the results of the 90-gene assay, the MDT adjudicated that the patient was diagnosed with a CUP/ breast cancer subset staged as T0N3M1. The patient who had been randomized to the EC arm received docetaxel plus cisplatin therapy with a PFS of two months and an OS of 15.6 months.

CASE 3

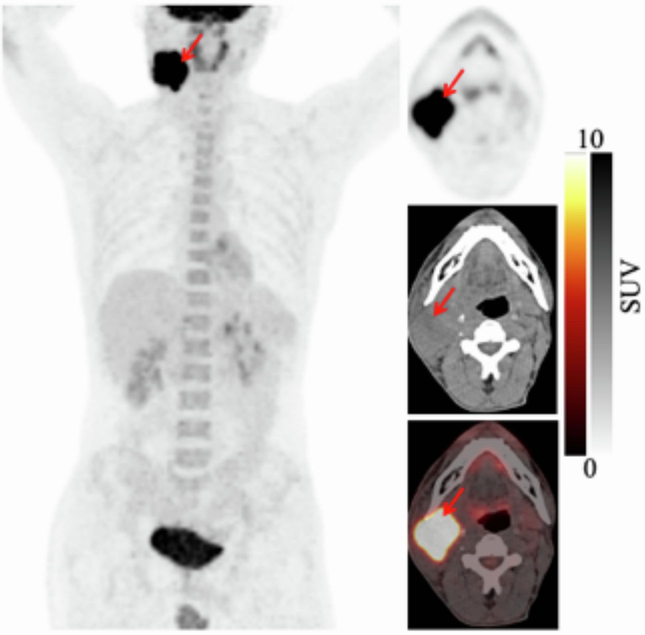

A 42-year-old man presented with enlarged right cervical lymph nodes for eight months. PET-CT scan demonstrated multiple lymph nodes with high ^{18}F-FDG uptake in the right neck, matching up with cancer metastasis. Other tests including laryngendoscope were unremarkable. Surgical biopsy indicated invasive metastatic poorly differentiated carcinoma with squamous-like differentiation, possibly poorly differentiated squamous cell carcinoma. IHC stains were negative for EBER, CK7, CK20, CDX2, AR, GATA3, PAX8 and TTF1, but positive for AE1/AE3, P40, CK5/6 and P16. The 90-gene assay showed a similarity score of 52.8% for head and neck squamous cell carcinoma. Based on results from pathology plus IHC and sentinel nodal theory (the right cervical lymph nodes), and incorporating the results of the 90-gene assay, the MDT adjudicated that the diagnosis of this patient was a CUP/ head and neck squamous cell carcinoma subset staged as T0N2M0. The patient who had been randomized to the EC arm was treated with gemcitabine plus cisplatin, with a PFS of 1.9 months and an OS of 22.2 months.

CASE 4

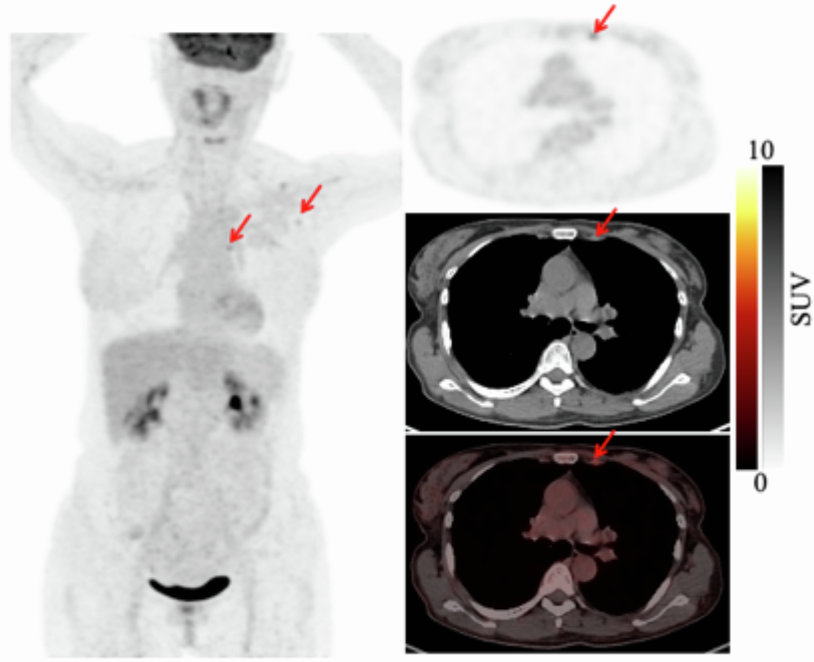

A 56-year-old woman presented with a mass in the left chest wall for two months. Breast MRI only revealed several irregular nodules within the left chest wall musculature. The patient underwent resection of the chest wall mass. Post-operative pathology of the mass indicated poorly differentiated carcinoma. IHC stains were negative for ER, PR, HER2, CK20, GATA3, GCDFP15, mammaglobin, PAX8, Arg1, inhibin, SF1 and TTF1, but positive for AE1/AE3, CK, CK7, CK5/6 and SOX10. The pathology and IHC results suggested a possible breast origin. Post-operative PET-CT scan demonstrated increased ^{18}F-FDG uptake in the intermuscular spaces of the chest wall, and the internal mammary lymph nodes. The 90-gene assay indicated a similarity score of 56.6% for breast cancer. NGS detected mutations in genes such as BRCA2, FGFR2 and PTEN. Drawing on the results from pathology plus IHC, the sentinel nodal theory (the internal mammary lymph node), and taking into account the outcomes of NGS and the 90-gene assay, the MDT adjudicated that the diagnosis of this patient was a CUP/ breast cancer subset staged as T0N2M1. The patient who had been randomized to the SST arm was treated with albumin-bound paclitaxel plus cisplatin, with a PFS of 33.3 months and an OS of 36.3 months.

CASE 5

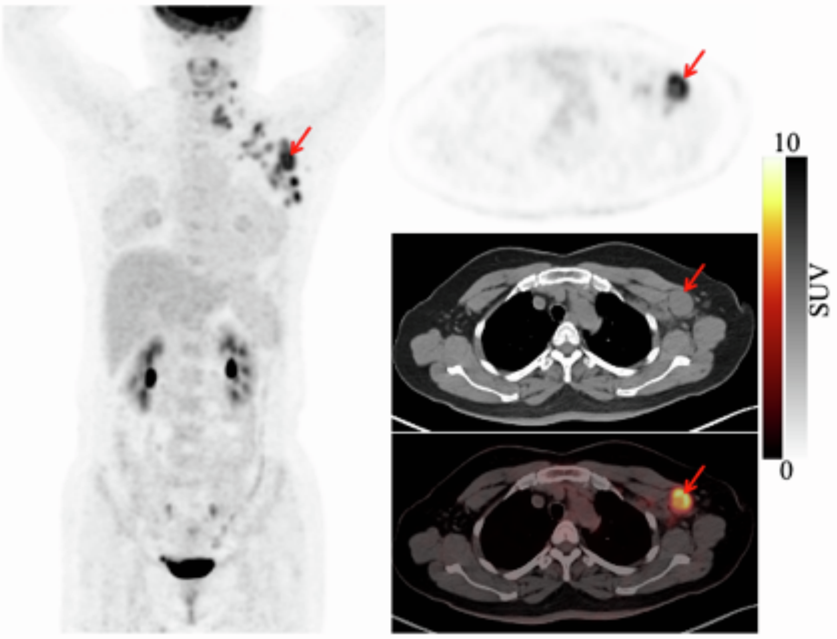

A 52-year-old woman presented with enlarged lymph nodes in the left neck and axilla. Physical examination was otherwise unremarkable. PET-CT scan demonstrated increased ^{18}F-FDG uptake in lymph nodes in the left neck, supraclavicular area, axilla and Rotter lymph nodes. Breast MRI only indicated enlarged left axillary and Rotter lymph nodes. Biopsy of the left cervical lymph nodes demonstrated poorly differentiated adenocarcinoma. IHC stains were negative for ER, HER2, mammaglobin, GCDFP15, GATA3, CK20, CDX2, P63, WT1, PAX8 and TTF1, but positive for CK7. Pathology and IHC results were incapable of determining the site of origin. The 90-gene assay indicated a similarity score of 88.7% for breast cancer. NGS detected mutations in genes like BRCA1, CDK12, and TP53. Based on results from sentinel nodal theory (the axillary and Rotter lymph nodes), as well as the outcomes of NGS and the 90-gene assay, the MDT adjudicated that the diagnosis of this patient was a CUP/ breast cancer subset staged as T0N3M1. The patient who had been randomized to the SST arm was treated with albumin-bound paclitaxel plus cisplatin, followed by olaparib maintenance with a PFS of 30.4 months and an OS of 31.5 months.

CASE 6

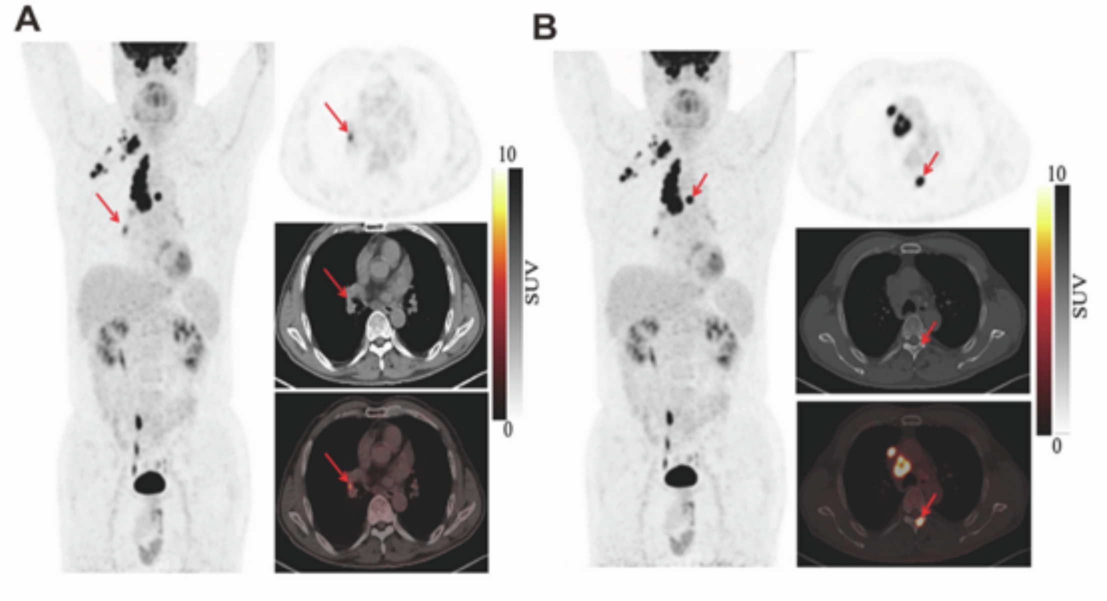

A 65-year-old man presented with enlarged lymph nodes in the right neck for one month. Physical examination was otherwise unremarkable. PET-CT scan demonstrated elevated ^{18}F-FDG uptake in the right lower cervical, supraclavicular, axillary, mediastinal and right hilar lymph nodes, as well as in the fifth thoracic vertebra (T5). Biopsy of the right cervical lymph node revealed poorly-differentiated carcinoma, with a tendency towards adenocarcinoma. IHC stains were negative for TTF1, CK20, PAX8, GATA3, P40, NKX3.1, CDX2, CgA and Syn, but positive for AE1/AE3, CK7 and CK19. The pathology and IHC results were incapable of determining the site of origin. The 90-gene assay indicated a similarity score of 61.7% for pulmonary origin. NGS indicated mutations in genes of KRAS and PIK3CA. Based on results from sentinel nodal theory (the right hilar lymph nodes), Batson plexus (the fifth thoracic vertebra) and the 90-gene assay, the MDT adjudicated that the diagnosis of this patient was a CUP/ lung cancer subset staged as T0N3M1. The patient who had been randomized to the SST arm was treated with pemetrexed, carboplatin and sintilimab with a PFS of 27.4 months and an OS of 31.9 months.

CASE 7

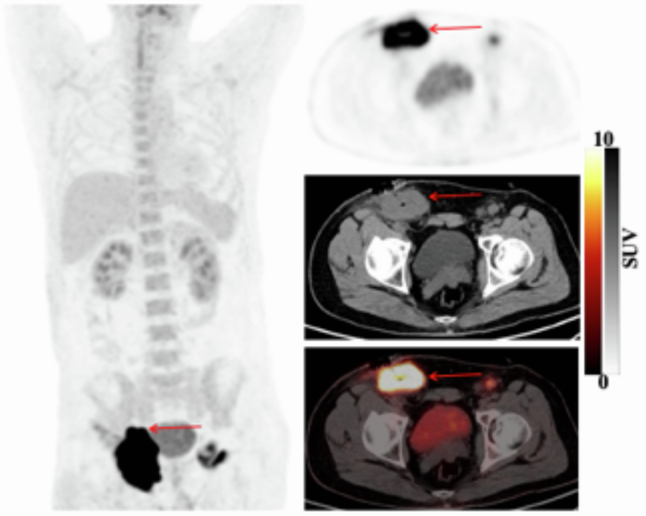

A 40-year-old man presented with a continuously enlarged subcutaneous mass in the right groin for nine months. Physical examination was otherwise unremarkable. PET-CT scan demonstrated elevated ^{18}F-FDG uptake within the mass in right inguinal region and lymph nodes in left inguinal regions. Biopsy of the right inguinal mass showed squamous cell carcinoma. IHC stains were negative for EBER, TTF1, PAX8, GATA3, CK20, P16 and CK7, but positive for AE1/AE3 and P63. The pathology and IHC results suggested squamous differentiation but could not indicate the site of origin. The 90-gene assay showed a similarity score of 67.4% for head and neck squamous cell carcinoma. Based on results from the pathology and sentinel nodal theory (the right lymph nodes in the groin), the MDT adjudicated that the diagnosis of this patient was a CUP/ squamous cell carcinoma of the perineal region or right leg staged as T0N2M0. The patient who had been randomized to the EC arm received gemcitabine plus cisplatin therapy with a PFS of nine months and an OS of 22.6 months.

CASE 8

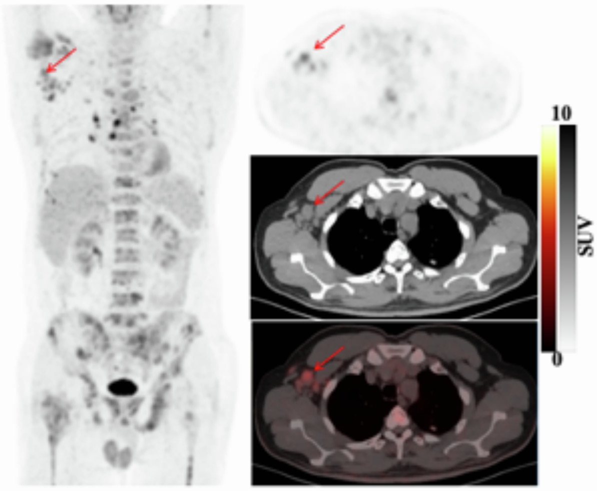

A 57-year-old man presented with pain in his right lower leg for six months. Physical examination was otherwise unremarkable. PET-CT scan demonstrated elevated ^{18}F-FDG uptake in multiple bones and the right axillary lymph nodes. Biopsy of the right axillary lymph nodes revealed metastatic poorly-differentiated adenocarcinoma. IHC stains were negative for GATA3, CK20, P63, TTF1, Hep1, and ER, but positive for GCDFP15, AR, mammaglobin and CK7. HER2 was 2+ but FISH negative.The pathology and IHC results suggested primary tumor site possibly being the breast or accessory mammary gland. The 90-gene assay showed a similarity score of 79.3% for breast cancer. Breast MRI was unremarkable. Based on results from the pathology plus IHC and sentinel nodal theory (the right axillary lymph nodes), and taking into account the outcomes of the 90-gene assay, the MDT adjudicated that the diagnosis of this patient was a CUP/ breast cancer staged as T0N1M1. The patient who had been randomized to the SST arm received albumin-bound paclitaxel plus cisplatin therapy with a PFS of 10.5 months and an OS of 23.8 months.

CASE 9

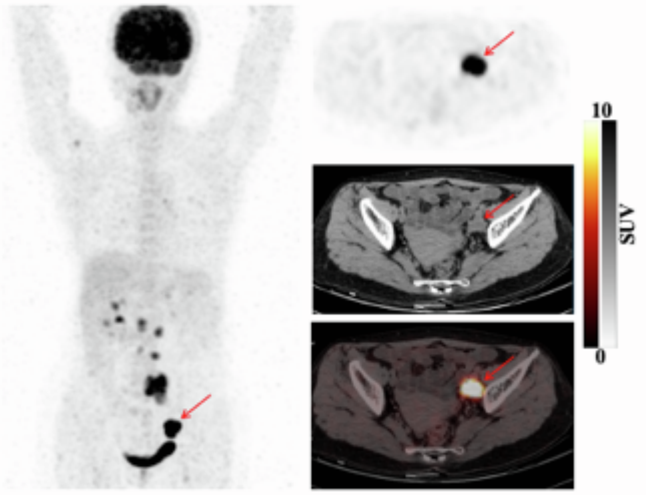

A 63-year-old woman presented with a dull soreness in the lumbosacral region for two years, which had worsened in the past month. Physical examination was otherwise unremarkable. PET-CT scan demonstrated elevated ^{18}F-FDG uptake in the nodule near the middle-upper segment of the left ureter and the lymph nodes adjacent to the left iliac vessels. Biopsy of the nodule revealed poorly-differentiated carcinoma. IHC stains were negative for P40, TTF1, CD56, CK20, CD20, PSA, CD56, Syn, CgA, P16, WT1, PAX-8, ER, PR, CDX2, M-CEA, SATB2, mammaglobin, GCDFP15, HER2 and GATA3, but positive for CK, CK7, vim, P53 and CK19. The Pathology and IHC results were incapable of determining the site of origin. The 90-gene assay showed a similarity score of 56.5% for breast cancer. Breast mammography was unremarkable. Although the patient had sentinel lymph nodes (the lymph nodes adjacent to the left iliac vessels), there was a lack of additional supporting evidence. Due to the conflicting situations among the 90-gene assay and sentinel lymph node, the MDT was incapable of adjudicating the diagnosis for this patient. The patient who had been randomized to the SST arm received gemcitabine plus cisplatin therapy with a PFS of 12.1 months and an OS of 49.5 months.

CASE 10

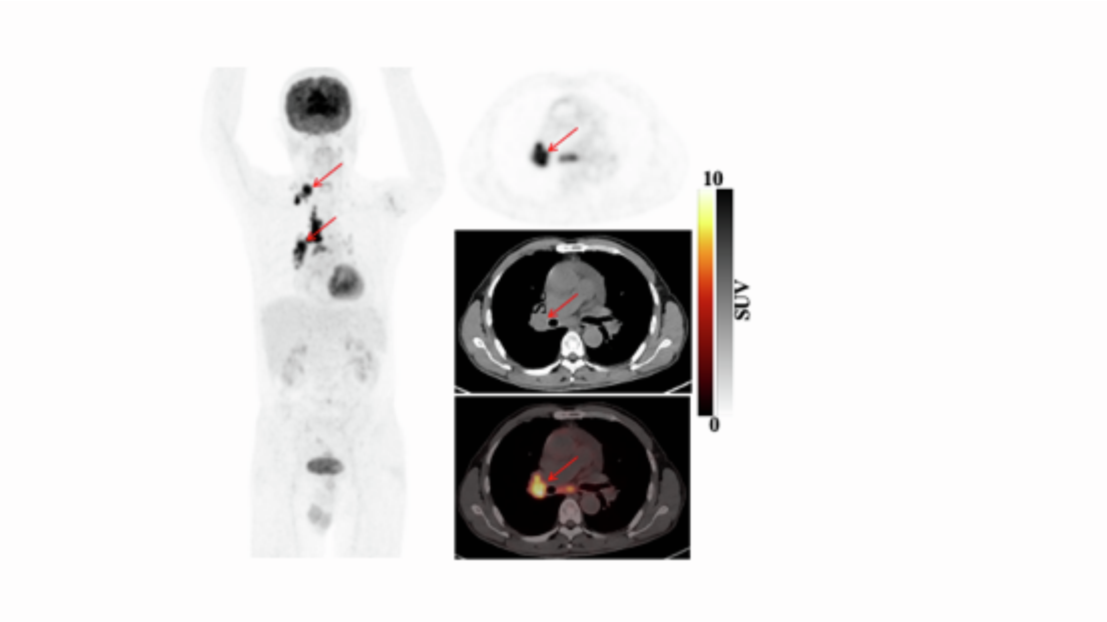

A 63-year-old man presented with enlarged lymph nodes in the right neck for nine months. Physical examination was otherwise unremarkable. PET-CT scan demonstrated elevated ^{18}F-FDG uptake in the right hilar, mediastinal and bilateral supraclavicular fossa lymph nodes, matching up with cancer metastasis. Biopsy of the lymph nodes in the right neck revealed metastatic adenocarcinoma. IHC stains were negative for Inhibin, Syn, CDX2-88, CK20, SF1, P40, TG, PAX8, AR, TTF1, Napsin A, GCDFP15, and Melan-A(A103), but positive for CK7. The pathology and IHC results did not suggest tissue of origin. The 90-gene assay showed a similarity score of 62.3% for urinary carcinoma. Although the patient had sentinel lymph nodes (the right hilar and mediastinal lymph nodes), there was a lack of additional supporting evidence. Consequently, the MDT was incapable of adjudicating the diagnosis for this patient. The patient who had been randomized to the EC arm received paclitaxel plus carboplatin therapy with a PFS of 4.8 months and an OS of 49.7 months.

CASE 11

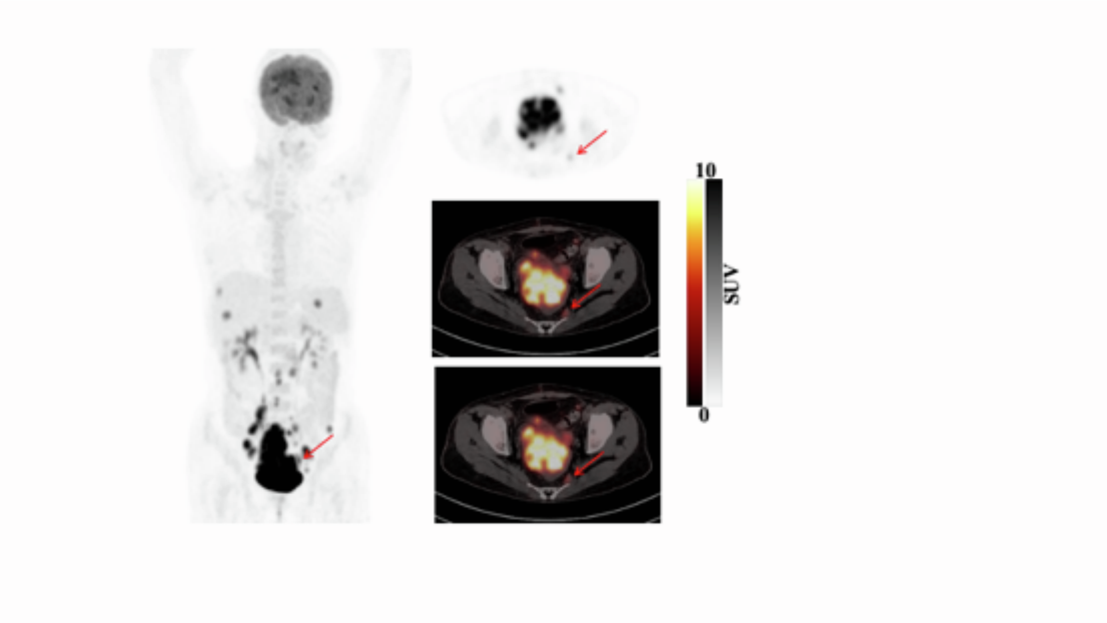

A 51-year-old woman presented with a pelvic mass for two months. The patient underwent resection of the mass along with the removal of the uterus and bilateral adnexa. Post-operative athology of the pelvic mass revealed metastatic adenocarcinoma. IHC stains were negative for CK20, CDX2, SATB2, PAX8, ER, and PR, but positive for CK7, P16, AE1/AE3, and SMAD4, suggesting the putative primary lesion being the stomach and pancreaticobiliary system. Post-op PET-CT scan demonstrated elevated ^{18}F-FDG uptake in multiple lymph nodes in the pre-sacral, right internal iliac, and retroperitoneal regions. EGD and colonoscopy showed no lesions. The 90-gene assay showed a similarity score of 18.3% for cervical cancer, ranking first. Based on results from the sentinel nodal theory (the pre-sacral lymph node) and the 90-gene assay, the MDT adjudicated that the diagnosis of this patient was a CUP/ cervical cancer staged as T0N1M1. The patient who had been randomized to the SST arm didn't receive any anti-tumor treatment and experienced a rapid disease progression 18 days later and an OS of 9.1 months.

CASE 12

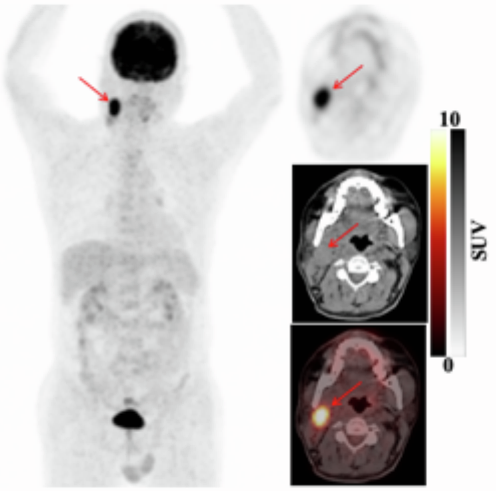

A 57-year-old man presented with enlarged lymph nodes in the right neck for 10 months. Physical examination was otherwise unremarkable. PET-CT scan demonstrated elevated ^{18}F-FDG uptake in the right cervical lymph node. Other tests including nasopharyngeal endoscope were unremarkable. Biopsy of the lymph node in the right neck revealed metastatic poorly differentiated squamous cell carcinoma. IHC stains were negative for CK7, TTF1, and TG, but positive for P40, CK5/6, PCK, P63, and EBER. The pathology and IHC results indicated squamous cell carcinoma. The 90-gene assay showed a similarity score of 56.2% for head and neck squamous cell carcinoma. Based on results from the sentinel nodal theory (the right cervical lymph node) and the 90-gene assay, the MDT adjudicated that the diagnosis of this patient was a CUP/ head and neck squamous cell carcinoma staged as T0N1M0. The patient who had been randomized to the EC arm received gemcitabine plus cisplatin therapy with a PFS of 2.8 months and an OS of 2.8+ months.

CASE 13

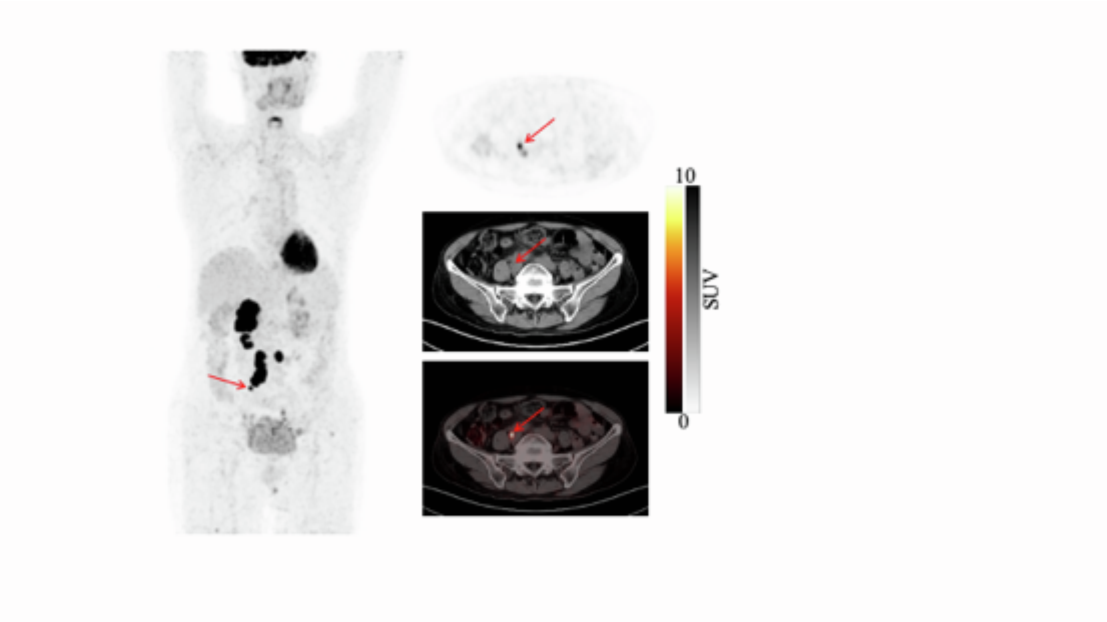

A 63-year-old woman presented with retroperitoneal mass for three months. Physical examination was unremarkable. PET-CT scan demonstrated elevated ^{18}F-FDG uptake in the retroperitoneal lymph nodes and the lymph nodes beside the iliac vessels. Biopsy of the retroperitoneal mass revealed metastatic poorly differentiated squamous cell carcinoma. IHC stains were negative for CK7, CK20, PAX8, Syn, CgA, Villin, CDX2, TTF1, CA125, ER, PR, GATA3, and napsin A, but positive for CKpan, P40, P16 and P63. The pathology and IHC results exhibited squamous differentiation but were incapable of determining the site of origin. The 90-gene assay showed a similarity score of 25.2% for cervical cancer, ranking first. Colonoscopy was unremarkable and cervical pathology suggested chronic cervicitis with squamous metaplasia. NGS indicated mutations in gene of FBXW7. Based on results from the sentinel nodal theory (the para-iliac lymph nodes) and the 90-gene assay, the MDT adjudicated that the diagnosis of this patient was a CUP/ cervical cancer staged as T0N1M1. The patient who had been randomized to the SST arm received cisplatin, paclitaxel and bevacizumab therapy with a PFS of 12.4 months and an OS of 14.1 months.

CASE 14

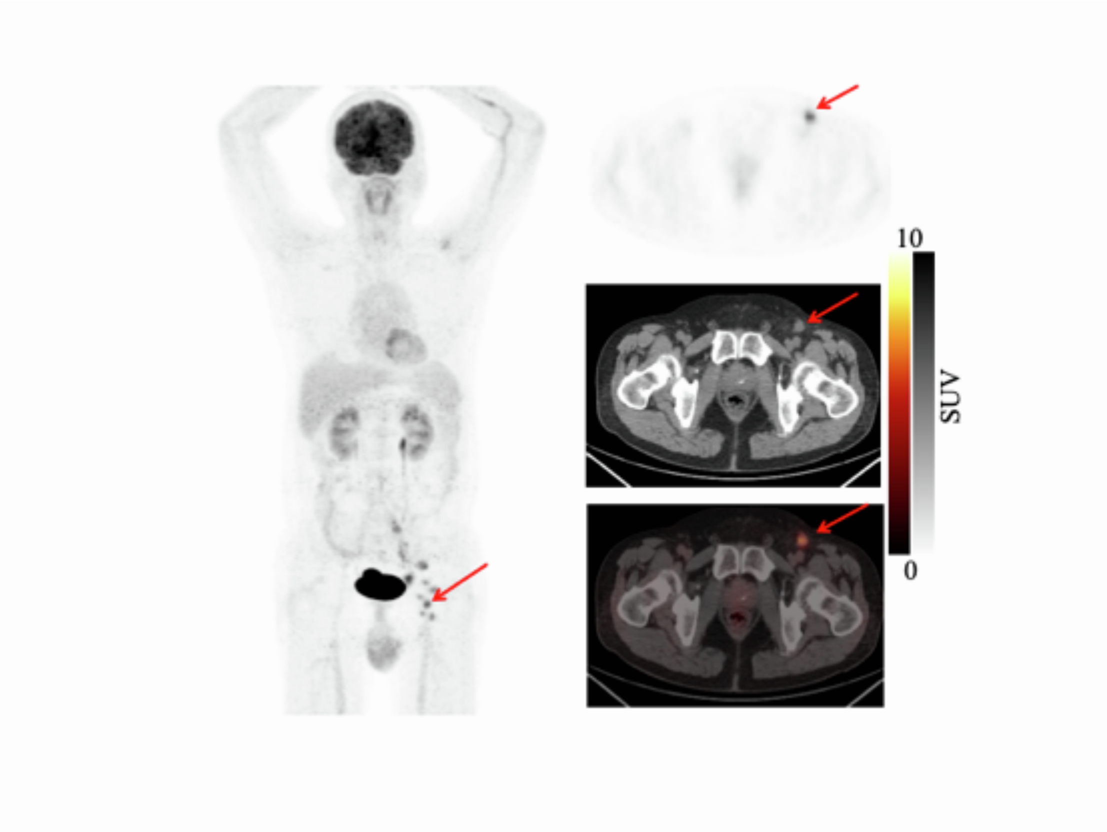

A 62-year-old man presented with enlarged lymph nodes in the left groin. Physical examination was otherwise unremarkable. EGD and colonoscopy showed no lesions. PET-CT scan demonstrated increased ^{18}F-FDG uptake in multiple lymph nodes in the retroperitoneal, pelvic, and left groin. Biopsy of the left inguinal lymph nodes revealed metastatic poorly differentiated carcinoma. IHC stains were negative for TTF1, PAX8, CK7, CK20, P40, NKX3.1 and PD-1, but positive for PMS2, hMSH2, hMSH6, hMLH-1, PD-L1, and AE1/AE3. The pathology and IHC results did not indicate the tissue origin. The 90-gene assay showed a similarity score of 25.9% for head and neck squamous cell carcinoma. Based on results from the pathology plus IHC and sentinel nodal theory (the left inguinal lymph nodes), the MDT adjudicated that the diagnosis of this patient was a CUP/ squamous cell carcinoma of the perineal region or left leg staged as T0N3M0. The patient who had been randomized to the SST arm was treated with gemcitabine plus cisplatin, with a PFS of 14.8 months and an OS of 52.9 months.

CASE 15

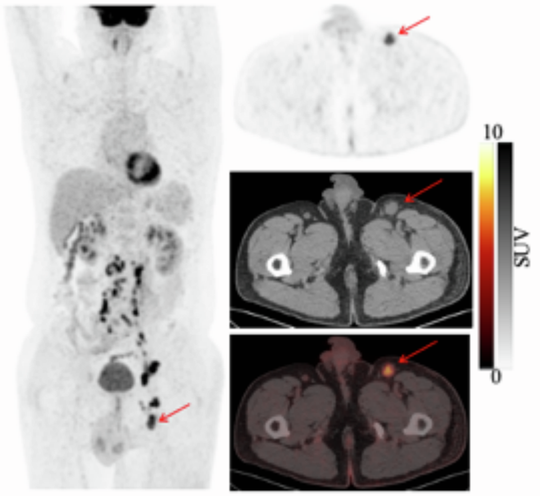

A 60-year-old man presented with enlarged lymph nodes in the left groin. Physical examination was otherwise unremarkable. PET-CT scan showed increased ^{18}F-FDG uptake in the retroperitoneal, pelvic and left inguinal lymph nodes. Biopsy of the left inguinal lymph nodes revealed metastatic poorly differentiated carcinoma. IHC stains were negative for EBER, TTF-1, ALK, Napsin A, CDX2, CK20, and NKX3.1, but positive for p40, CK7, PD-L1 (TPS 20%), and p63, with ambiguous expressions of GATA3 and PAX8. The pathology and IHC results suggested poorly differentiated squamous cell carcinoma, not suggestive of tissue of origin. The 90-gene assay showed a similarity score of 29.2% for head and neck squamous cell carcinoma, ranking first. Based on results from the pathology plus IHC and sentinel nodal theory (the left inguinal lymph nodes), the MDT adjudicated that the diagnosis of this patient was a CUP/ squamous cell carcinoma of the perineal region or left leg staged as T0N3M1. The patient who had been randomized to the SST arm was treated with albumin-bound paclitaxel, cisplatin and sintilimab, with a PFS of 20 months and an OS of 26.3 months.

CASE 16

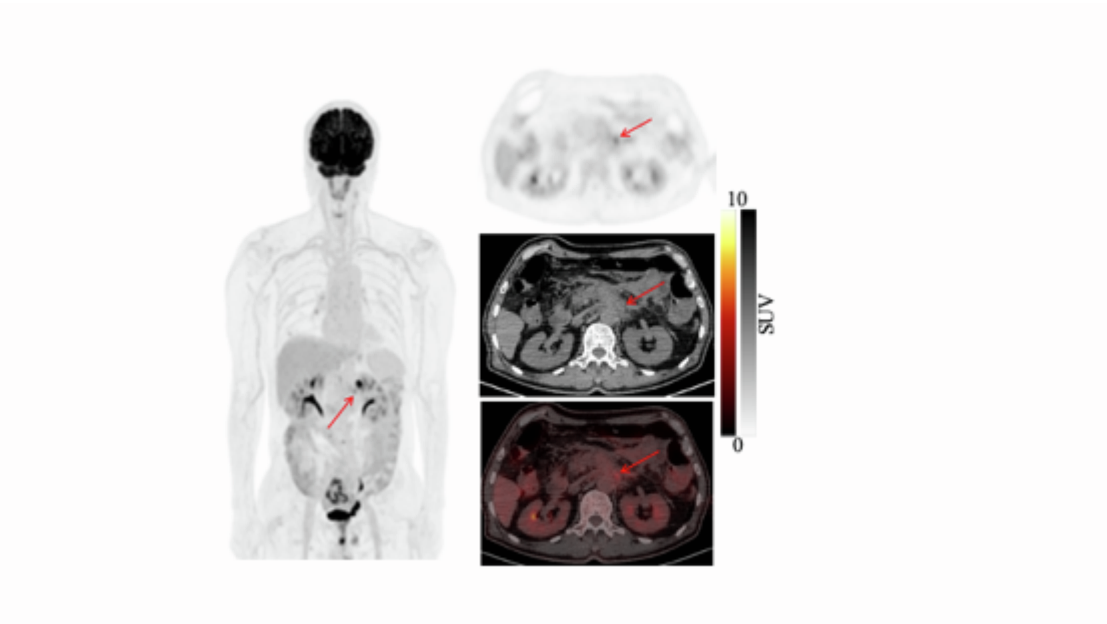

A 66-year-old man presented with diarrhea for seven months. Physical examination was unremarkable. EGD and colonoscopy showed no lesions. PET-CT scan demonstrated increased ^{18}F-FDG uptake in the retroperitoneal lymph node and the left adrenal gland. The patient underwent resection of the left adrenal node. Post-operative pathology of the adrenal node revealed metastatic adenocarcinoma. IHC stains were negative for TTF1, PAX8,CK20,CDX2-88, but positive for CK7, CK19, MUC1, and SMAD4, suggesting the putative primary lesion being the pancreas or biliary tract. The 90-gene assay showed a similarity score of 38.3% for pancreatic cancer. Based on results from the pathology plus IHC, sentinel nodal theory (the retroperitoneal lymph node) and the 90-gene assay, the MDT adjudicated that the diagnosis of this patient was a CUP/ pancreatic cancer staged as T0N1M1. The patient who had been randomized to the SST arm was treated with gemcitabine plus nab-paclitaxel, with a PFS of 9.8 months and an OS of 22.1 months.

CASE 17

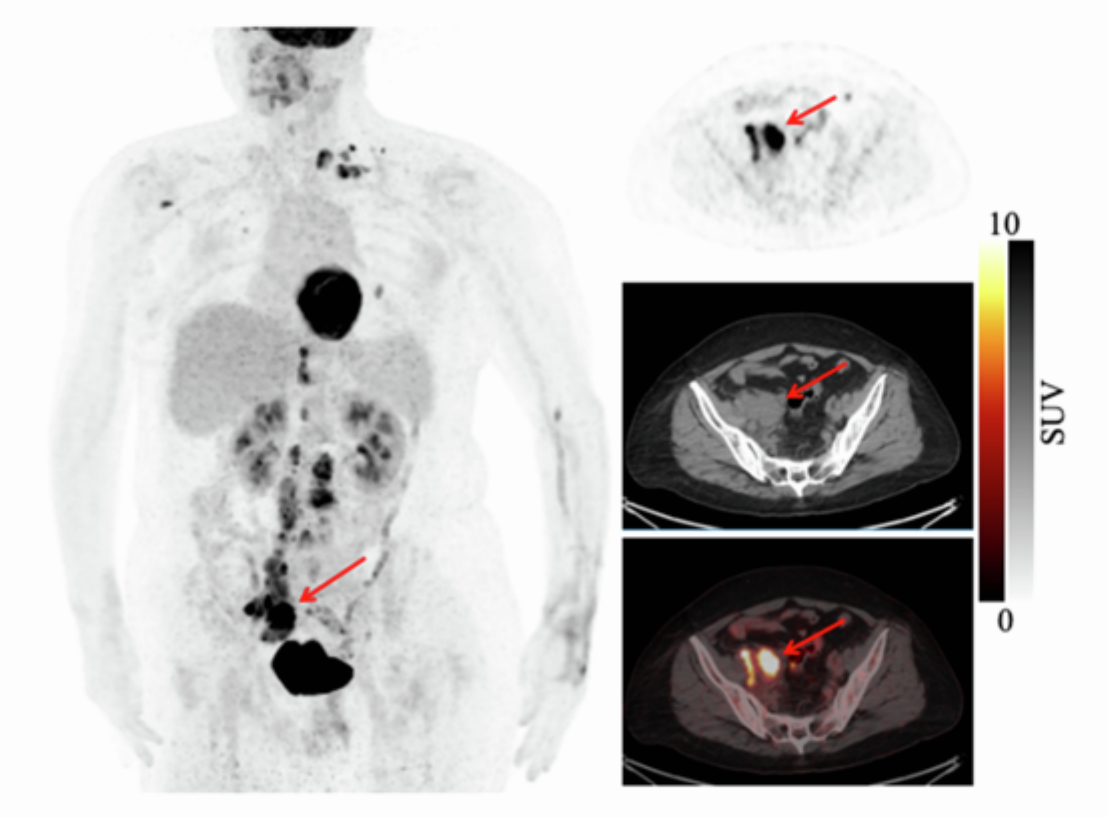

A 53-year-old woman presented with lower abdominal distension accompanied by right lower limb edema for four months. Physical examination disclosed enlarged lymph nodes in the left neck and supraclavicular region. PET-CT scan demonstrated elevated ^{18}F-FDG uptake in the right adnexal area, left supraclavicular, retroperitoneal and right para-iliac lymph nodes, bilateral lungs and L3 vertebral body, consistent with cancer metastases. Biopsy of the left supraclavicular lymph nodes revealed metastatic squamous cell carcinoma. The patient underwent laparoscopic exploration and conization of the uterine cervix. Biopsy of the uterine cervix showed no evidence of malignancy. Biopsy of the right pelvic lymph nodes revealed poorly-differentiated squamous cell carcinoma. IHC stains were negative for TTF1, ER, PR, PAX8, WT1, CDX2, and CK20, but positive for P40, P63, GATA3, CK5/6, CK7, P16, P53 and ki67 (50%+). The pathology and IHC results exhibited squamous differentiation but did not suggest tissue of origin. The 90-gene assay showed a similarity score of 92.2% for cervical cancer. Based on results from the sentinel nodal theory (the right para-iliac lymph nodes) and the 90-gene assay, the MDT adjudicated that the diagnosis of this patient was a CUP/ cervical cancer staged as T0N1M1. The patient who had been randomized to the SST

arm received cisplatin plus paclitaxel therapy with a PFS of 9.5 months and an OS of 24.1 months.

Appendix 2. Batson plexus theory

CASE 1

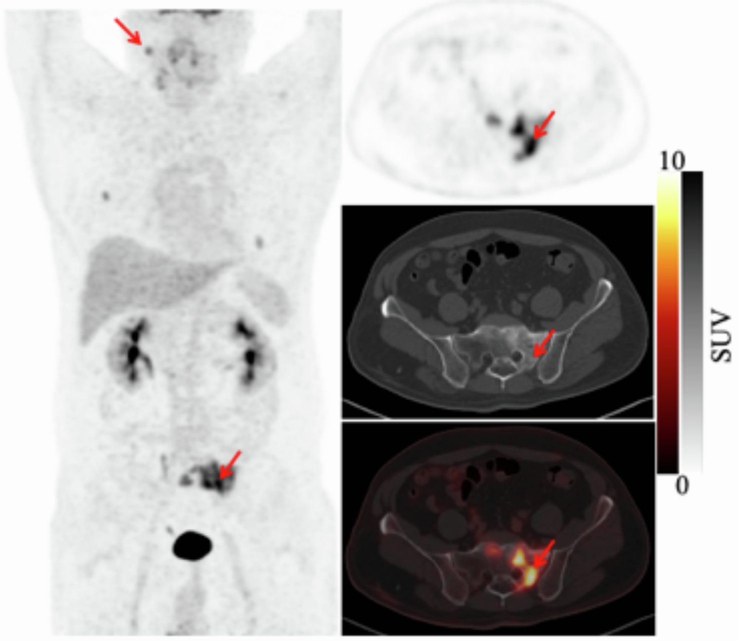

A 49-year-old man presented with a CEA elevation for more than two months. PET-CT scan demonstrated increased ^{18}F-FDG uptake in lymph nodes in the right deep cervical space and parapharyngeal space, in bilateral pulmonary nodules, along with the first sacral vertebra (S1) metastasis. Other tests including laryngoscope were unremarkable. Biopsy of the left sacroiliac joint revealed metastatic carcinoma, with a tendency towards adenocarcinoma. IHC stains were negative for PAX8, CK20, P63, TTF1, ER and PR, but positive for GATA3, AR, CK7, AE1/AE3, GCDFP15, and HER2. Pathology and IHC results indicated apocrine differentiation, consistent with cancer of breast origin. The 90-gene assay indicated a similarity score of 65.7% for breast cancer. Based on results from pathology plus IHC and the 90-gene assay, the MDT adjudicated that the diagnosis of this patient was a CUP/ breast cancer subset staged as T0N3M1. The patient who had been randomized to the SST arm was treated with liposomal paclitaxel, cisplatin, and trastuzumab with a PFS of 10.1 months and an OS of 45.7 months.

CASE 2

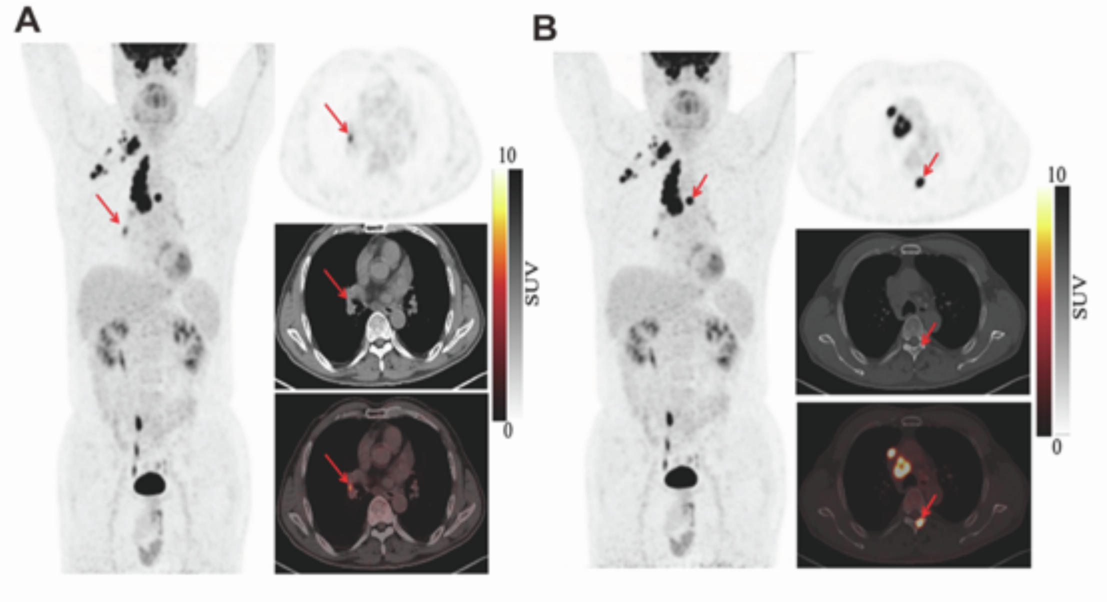

A 65-year-old man presented with enlarged lymph nodes in the right neck for one month. Physical examination was otherwise unremarkable. PET-CT scan demonstrated elevated ^{18}F-FDG uptake in the right lower cervical, supraclavicular, axillary, mediastinal and right hilar lymph nodes, as well as in the fifth thoracic vertebra (T5). Biopsy of the right cervical lymph node revealed poorly-differentiated carcinoma, with a tendency towards adenocarcinoma. IHC stains were negative for TTF1, CK20, PAX8, GATA3, P40, NKX3.1, CDX2, CgA and Syn, but positive for AE1/AE3, CK7 and CK19. Pathology and IHC results were incapable of precisely determining the site of origin. The 90-gene assay indicated a similarity score of 61.7% for pulmonary origin. NGS indicated mutations in genes of KRAS and PIK3CA. Based on results from Batson plexus (the fifth thoracic vertebra) and the 90-gene assay, the MDT adjudicated that the diagnosis of this patient was a CUP/ lung cancer subset staged as T0N3M1. The patient who had been randomized to the SST arm was treated with pemetrexed, carboplatin and sintilimab with a PFS of 27.4 months and an OS of 31.9 months.

CASE 3

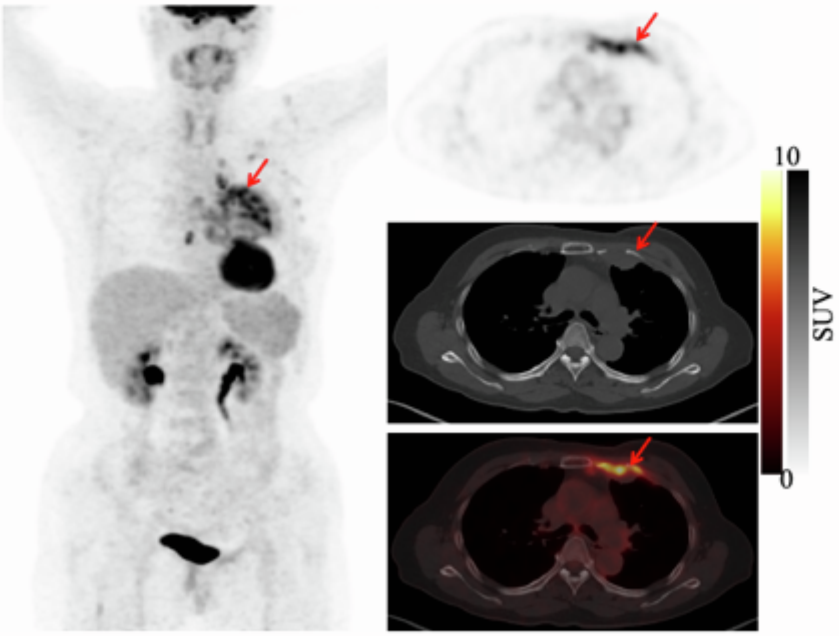

A 59-year-old woman presented with a chest wall mass for two months. Physical examination was otherwise unremarkable. PET-CT scan demonstrated elevated ^{18}F-FDG uptake in the left chest wall, the sternum, the second and third ribs. Post-operative pathology of the left chest wall mass revealed poorly-differentiated carcinoma. IHC stains were negative for ER, HER2, mamaglobin, CK20, CDX-2, PAX8, WT1, Calretinin and D2-40, but positive for AE1/AE3, CK5/6, CK7, SMARCA4 BRG1, SMARCB1(INI-1) and Ki-67 （80%+）. Pathology and IHC results were incapable of precisely determining the site of origin. The 90-gene assay indicated a similarity score of 52.3% for breast cancer. Based on results from Batson plexus (the second and third ribs) and the 90-gene assay, the MDT adjudicated that the diagnosis of this patient was a CUP/breast cancer subset staged as T0N0M1.The patient who had been randomized to the EC arm was treated with paclitaxel plus cisplatin with a PFS of 27.6 months and an OS of 27.6+ months.

Appendix 3. MDT revision of the 90-gene diagnosis

CASE 1

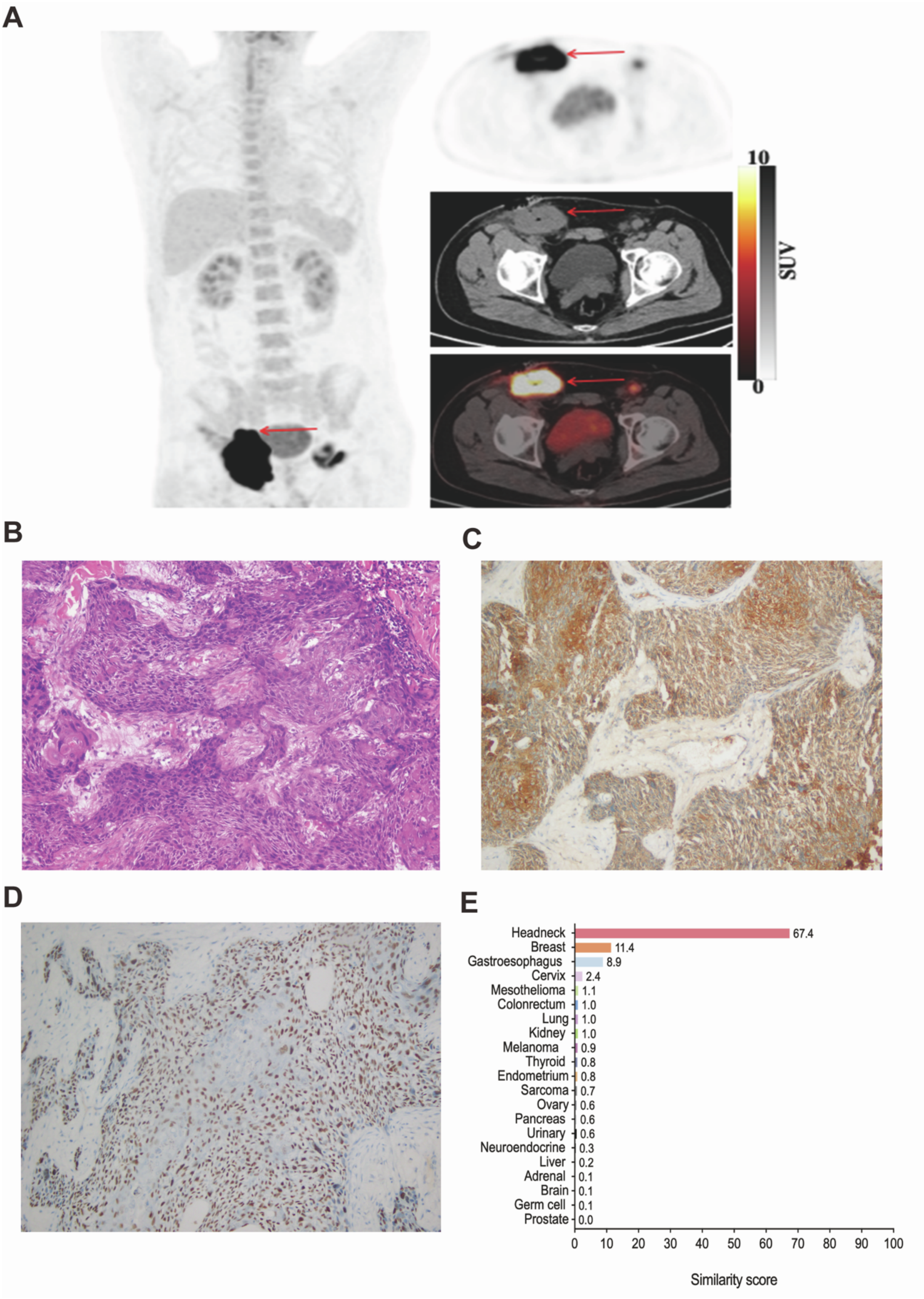

MDT in the management of cancer of unknown primary: diagnosis of presumptive primary tumors, TNM staging, and therapeutic strategies.

A 40-year-old man presented with a continuously enlarged subcutaneous mass in the right groin for

nine months. Physical examination was otherwise unremarkable. PET-CT scan demonstrated elevated ^{18}F-FDG uptake within the mass in right inguinal region and lymph nodes in left inguinal regions (**A**). Biopsy of the right inguinal mass showed squamous cell carcinoma (**B**). Biopsy of the right inguinal mass showed squamous cell carcinoma. IHC stains were negative for EBER, TTF1, PAX8, GATA3, CK20, P16 and CK7, but positive for AE1/AE3 (**C**) and P63 (**D**). The pathology and IHC results suggested squamous differentiation but could not indicate the site of origin. The 90-gene assay showed a similarity score of 67.4% for head and neck squamous cell carcinoma (**E**). Based on results from the pathology and sentinel nodal theory (the right lymph nodes in the groin, red arrow), the MDT adjudicated that the diagnosis of this patient was a CUP/ squamous cell carcinoma of the perineal region or right leg staged as T0N2M0. The patient who had been randomized to the EC arm received gemcitabine plus cisplatin therapy with a PFS of nine months and an OS of 22.6 months.

CASE 2

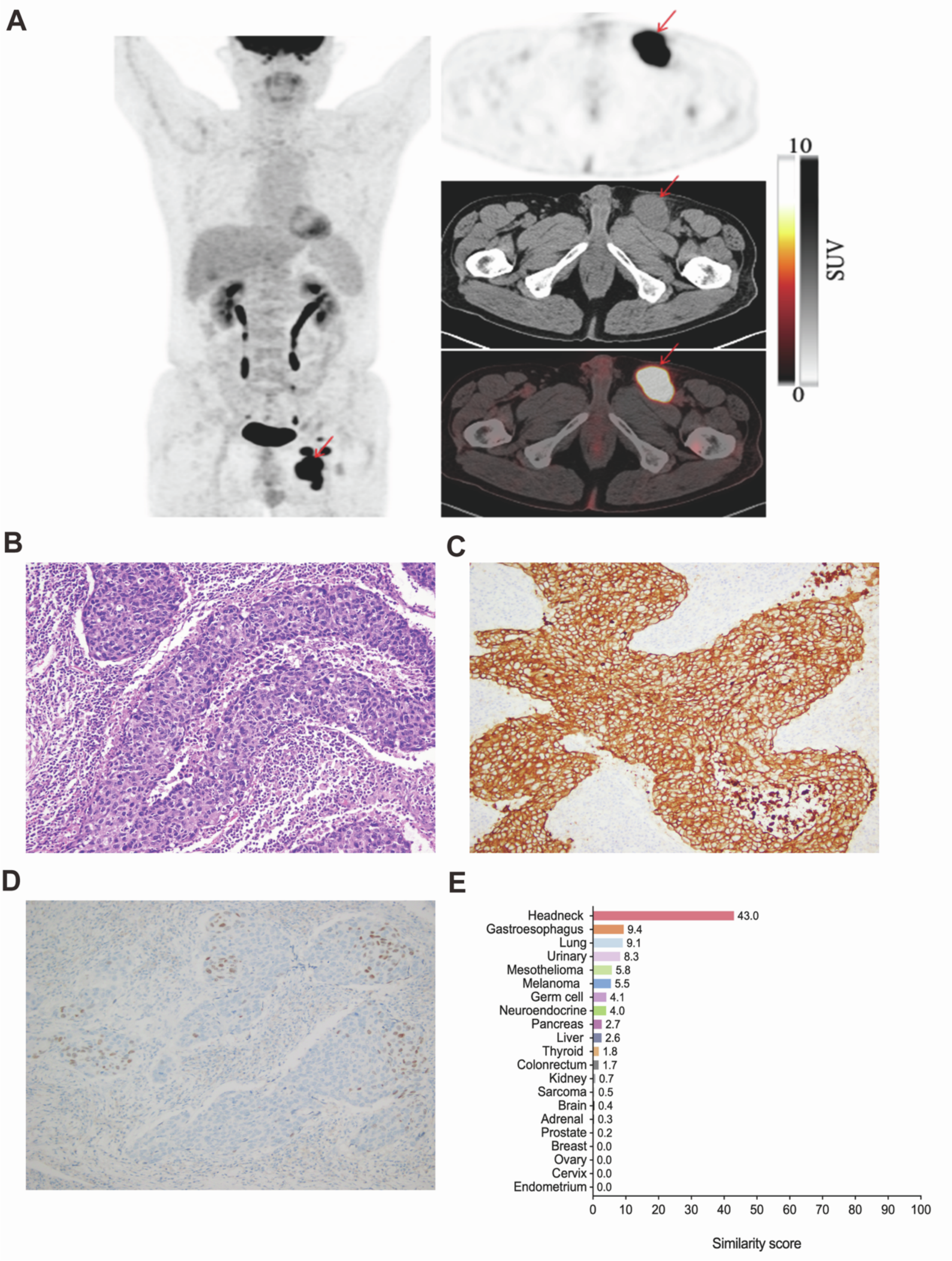

MDT in the management of cancer of unknown primary: diagnosis of presumptive primary tumors, TNM staging, and therapeutic strategies.

A 65-year-old man presented with continuously enlarged lymph nodes in the left groin for six months. Physical examination was otherwise unremarkable. PET-CT scan showed increased ^{18}F-

FDG uptake in the left inguinal and paravascular iliac lymph nodes, being consistent with cancer metastasis (**A**). Biopsy of the left inguinal lymph node revealed focal squamous differentiation (**B**). IHC stains were negative for GATA3, SOX10, HMB45, SALL4, Oct4, PSA, CDX2, Syn, CgA, CD56, AR, Arg-1, GPC-3 and EBER, but positive for AE1/AE3 (**C**), P40 (**D**), INI-1, and SMACA4. The pathology and IHC results did not suggest tissue of origin. The 90-gene assay showed a similarity score of 43% for head and neck squamous cell carcinoma (**E**). NGS identified mutations in ARID1A, EPHB1, KMT2C, PARP1, PIK3C2G, SMAD4, and TP53. Based on results from the pathology plus IHC and sentinel nodal theory (the left inguinal lymph node, red arrow), the MDT adjudicated that the diagnosis of this patient was a CUP/ squamous cell carcinoma of the perineal region or left leg staged as T0N1M0. The patient who had been randomized to the EC arm received gemcitabine plus cisplatin therapy with a PFS of 7.9 months and an OS of 37.7 months.

CASE 3

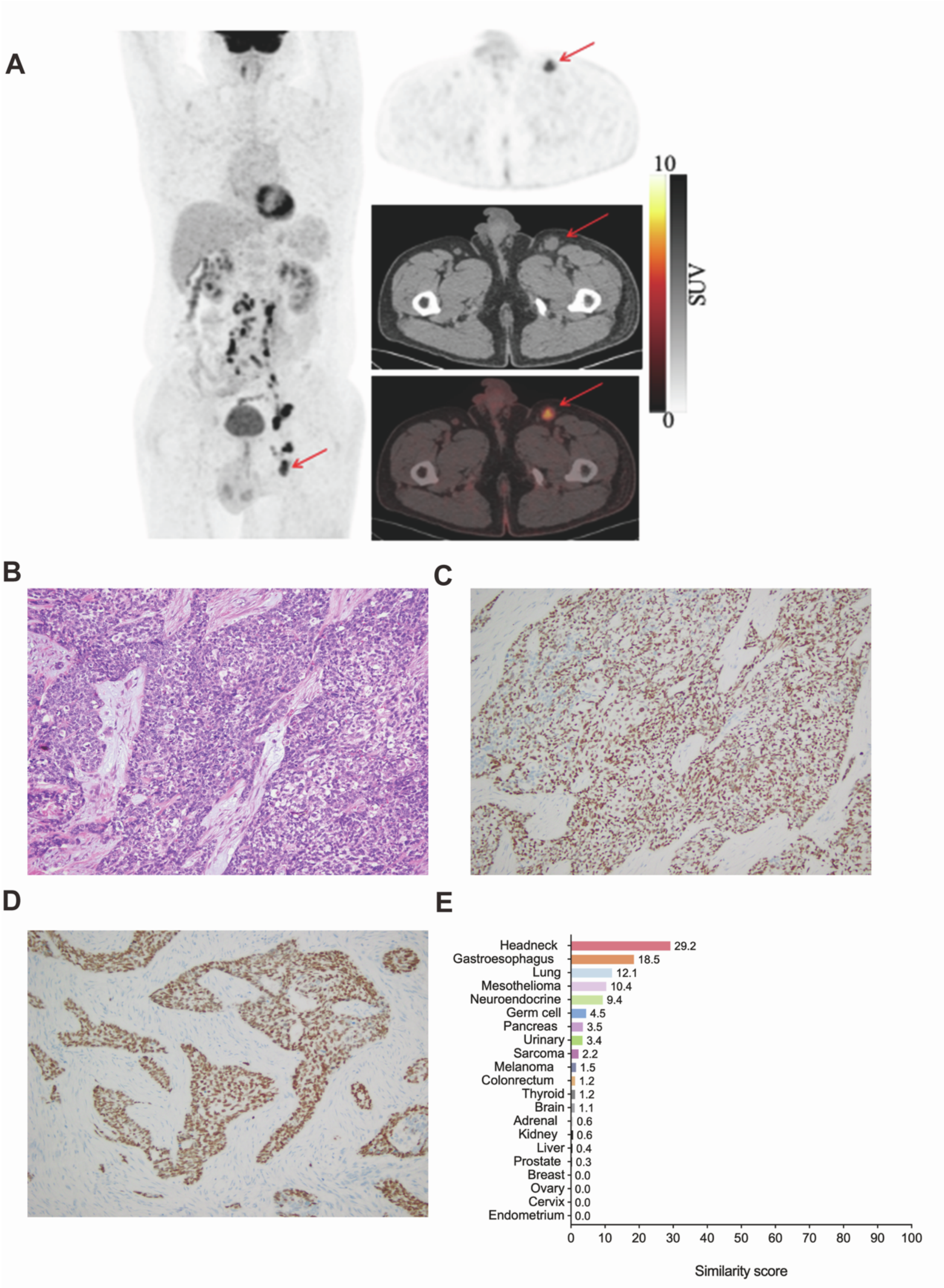

MDT in the management of cancer of unknown primary: diagnosis of presumptive primary tumors, TNM staging, and therapeutic strategies.

A 60-year-old man presented with enlarged lymph nodes in the left groin. Physical examination was otherwise unremarkable. PET-CT scan showed increased ^{18}F-FDG uptake in the retroperitoneal, pelvic and left inguinal lymph nodes (**A**). Biopsy of the left inguinal lymph nodes revealed metastatic poorly differentiated carcinoma (**B**). IHC stains were negative for EBER, TTF-1, ALK, Napsin A, CDX2, CK20, and NKX3.1, but positive for p40 (**C**), CK7, PD-L1 (TPS 20%), and p63 (**D**), with ambiguous expressions of GATA3 and PAX8. The pathology and IHC results suggested poorly differentiated squamous cell carcinoma, not suggestive of tissue of origin. The 90-gene assay showed a similarity score of 29.2% for head and neck squamous cell carcinoma, ranking first (**E**). Based on results from the pathology plus IHC and sentinel nodal theory (the left inguinal lymph nodes), the MDT adjudicated that the diagnosis of this patient was a CUP/ squamous cell carcinoma of the perineal region or left leg staged as T0N3M1. The patient who had been randomized to the SST arm was treated with albumin-bound paclitaxel, cisplatin and sintilimab, with a PFS of 20 months and an OS of 26.3 months.

CASE 4

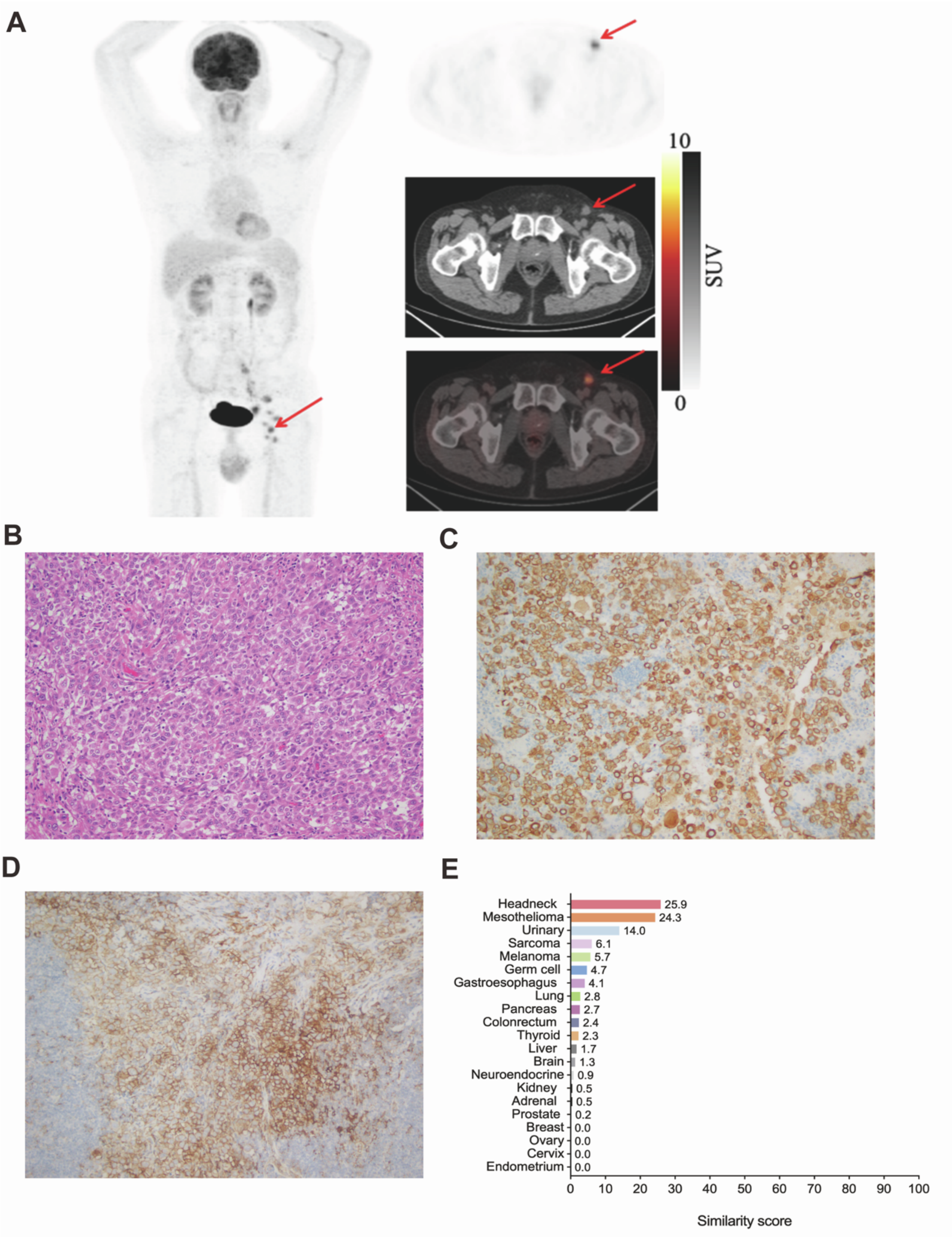

MDT in the management of cancer of unknown primary: diagnosis of presumptive primary tumors, TNM staging, and therapeutic strategies.

A 62-year-old man presented with enlarged lymph nodes in the left groin. Physical examination was otherwise unremarkable. Esophagogastroduodenoscopy (EGD) and colonoscopy showed no lesions. PET-CT scan demonstrated increased ^{18}F-FDG uptake in multiplelymph nodes in the retroperitoneal,

pelvic, and left groin (**A**). Biopsy of the left inguinal lymph nodes revealed metastatic poorly differentiated carcinoma (**B**). IHC stains were negative for TTF1, PAX8, CK7, CK20, P40, NKX3.1 and PD-1, but positive for PMS2, hMSH2, hMSH6, hMLH-1, AE1/AE3 (**C**) and PD-L1 (**D**). The pathology and IHC results did not indicate the tissue origin. The 90-gene assay showed a similarity score of 25.9% for head and neck squamous cell carcinoma (**E**). Based on results from the pathology plus IHC and sentinel nodal theory (the left inguinal lymph nodes, red arrow), the MDT adjudicated that the diagnosis of this patient was a CUP/ squamous cell carcinoma of the perineal region or left leg staged as T0N3M0. The patient who had been randomized to the SST arm was treated with gemcitabine plus cisplatin, with a PFS of 14.8 months and an OS of 52.9 months.

CASE 5

MDT in the management of cancer of unknown primary: diagnosis of presumptive primary tumors, TNM staging, and therapeutic strategies.

A 55-year-old man presented with left lumbosacral pain radiating to the left lower limb. Physical examination was otherwise unremarkable. EGD and colonoscopy showed no lesions. PET-CT scan demonstrated increased ^{18}F-FDG uptake in the superior mediastinal lymph nodes adjacent to the trachea, and left iliac bone. Biopsy of the left sacral iliac bone revealed metastatic poorly differentiated carcinoma, possibly adenocarcinoma. IHC stains were negative for CDX2, PAX8, CK20, PSA, NKX3.1, AR, TTF-1, P63, P40 and GATA3, but positive for CK7. The pathology and IHC results did not indicate the tissue origin. The 90-gene assay showed a similarity score of 31% for gastroesophageal origin. NGS revealed GATA6, RET, and ROS1mutations. Based on results from the sentinel nodal theory (the mediastinal lymph node), as well as the outcomes of NGS and the 90-gene assay, the MDT adjudicated that the diagnosis of this patient was a CUP/ lung or esophageal cancer subset staged as T0NxM1. The patient who had been randomized to the SST arm was treated with albumin-bound paclitaxel plus cisplatin, with a PFS of three months and an OS of 6.2 months.

References

1. Ye Q, Wang Q, Qi P, Chen J, Sun Y, Jin S, Ren W, Chen C, Liu M, Xu M, et al. Development and Clinical Validation of a 90-Gene Expression Assay for Identifying Tumor Tissue Origin. *J Mol Diagn*. 2020; **22**(9): 1139-1150.

2. Shao Y, Liu X, Hu S, Zhang Y, Li W, Zhou X, Wang Q, Hou Y, Chen Y, Wang Y, et al. Sentinel node theory helps tracking of primary lesions of cancers of unknown primary. *BMC Cancer*. 2020; **20**(1): 639.

3. Zhu M, Liu X, Qu Y, Hu S, Zhang Y, Li W, Zhou X, Yang H, Zhou L, Wang Q, et al. Bone metastasis pattern of cancer patients with bone metastasis but no visceral metastasis. *J Bone Oncol*. 2019; **15**: 100219.

4. Johnson P, Zhou Q, Dao DY, Lo YMD. Circulating biomarkers in the diagnosis and management of hepatocellular carcinoma. *Nat Rev Gastroenterol Hepatol*. 2022; **19**(10): 670-681.

5. Seckl MJ, Sebire NJ, Fisher RA, Golfier F, Massuger L, Sessa C, Group EGW. Gestational trophoblastic disease: ESMO Clinical Practice Guidelines for diagnosis, treatment and follow-up. *Ann Oncol*. 2013; **24 Suppl 6**: vi39-50.

6. Chang AJ, Autio KA, Roach M, 3rd, Scher HI. High-risk prostate cancer-classification and therapy. *Nat Rev Clin Oncol*. 2014; **11**(6): 308-323.

7. Xu Y, Zhang P, Zhang K, Huang C. The application of CA72-4 in the diagnosis, prognosis, and treatment of gastric cancer. *Biochim Biophys Acta Rev Cancer*. 2021; **1876**(2): 188634.

8. Luo G, Jin K, Deng S, Cheng H, Fan Z, Gong Y, Qian Y, Huang Q, Ni Q, Liu C, et al. Roles of CA19-9 in pancreatic cancer: Biomarker, predictor and promoter. *Biochim Biophys Acta Rev Cancer*. 2021; **1875**(2): 188409.

9. Shimada H, Noie T, Ohashi M, Oba K, Takahashi Y. Clinical significance of serum tumor markers for gastric cancer: a systematic review of literature by the Task Force of the Japanese Gastric Cancer Association. *Gastric Cancer*. 2014; **17**(1): 26-33.

10. Nilsson O, Johansson C, Glimelius B, Persson B, Norgaard-Pedersen B, Andren-Sandberg A, Lindholm L. Sensitivity and specificity of CA242 in gastro-intestinal cancer. A comparison with CEA, CA50 and CA 19-9. *Br J Cancer*. 1992; **65**(2): 215-

221.
11. Jiang XT, Tao HQ, Zou SC. Detection of serum tumor markers in the diagnosis and treatment of patients with pancreatic cancer. *Hepatobiliary Pancreat Dis Int*. 2004; **3**(3): 464-468.
12. Spechler SJ. Barrett esophagus and risk of esophageal cancer: a clinical review. *JAMA*. 2013; **310**(6): 627-636.
13. Chen YP, Chan ATC, Le QT, Blanchard P, Sun Y, Ma J. Nasopharyngeal carcinoma. *Lancet*. 2019; **394**(10192): 64-80.
14. Smith R. Paget's disease of bone. *BMJ*. 1992; **305**(6866): 1379-1380.
15. Piekarski J, Jeziorski A, Baklinska M, Szymczak W, Zadrozny M, Berner J. Patients with Paget disease of nipple and with palpable mass in breast have unfavorable prognosis. *J Exp Clin Cancer Res*. 2004; **23**(1): 33-37.
16. Mosele F, Remon J, Mateo J, Westphalen CB, Barlesi F, Lolkema MP, Normanno N, Scarpa A, Robson M, Meric-Bernstam F, et al. Recommendations for the use of next-generation sequencing (NGS) for patients with metastatic cancers: a report from the ESMO Precision Medicine Working Group. *Ann Oncol*. 2020; **31**(11): 1491-1505.
17. Rebello RJ, Oing C, Knudsen KE, Loeb S, Johnson DC, Reiter RE, Gillessen S, Van der Kwast T, Bristow RG. Prostate cancer. *Nat Rev Dis Primers*. 2021; **7**(1): 9.
18. French C. NUT midline carcinoma. *Nat Rev Cancer*. 2014; **14**(3): 149-150.
19. Kramer A, Bochtler T, Pauli C, Baciarello G, Delorme S, Hemminki K, Mileshkin L, Moch H, Oien K, Olivier T, et al. Cancer of unknown primary: ESMO Clinical Practice Guideline for diagnosis, treatment and follow-up. *Ann Oncol*. 2023; **34**(3): 228-246.
